# Efficient combinatorial adaptor-mediated targeting of acute myeloid leukemia with CAR T-cells

**DOI:** 10.1038/s41375-024-02409-1

**Published:** 2024-09-18

**Authors:** Laura Volta, Renier Myburgh, Christian Pellegrino, Christian Koch, Monique Maurer, Francesco Manfredi, Mara Hofstetter, Anne Kaiser, Florin Schneiter, Jan Müller, Marco M. Buehler, Roberto De Luca, Nicholas Favalli, Chiara F. Magnani, Timm Schroeder, Dario Neri, Markus G. Manz

**Affiliations:** 1 Institute of Pharmaceutical Sciences, Department of Chemistry and Applied Biosciences, ETH Zurich, Zurich, Switzerland; 2 Department of Medical Oncology and Hematology, University Hospital Zurich and University of Zurich, Zurich, Switzerland; 3 Department of Biosystems Science and Engineering, ETH Zurich, Basel, Switzerland; 4 Department of Pathology and Molecular Pathology, University Hospital Zurich, Zurich, Switzerland; 5 Philochem AG, Otelfingen, Switzerland; 6 Comprehensive Cancer Center Zurich (CCCZ), Zurich, Switzerland

**Keywords:** Immunotherapy, Acute myeloid leukaemia, Preclinical research

## Abstract

CAR T-cell products targeting lineage-specific cell-of-origin antigens, thereby eliminating both tumor and healthy counterpart cells, are currently clinically approved therapeutics in B- and plasma-cell malignancies. While they represent a major clinical improvement, they are still limited in terms of efficacy by e.g. single, sometimes low-expressed antigen targeting, and in terms of safety by e.g., lack of on-off activity. Successful cell-of-origin non-discriminative targeting of heterogeneous hematopoietic stem and progenitor cell malignancies, such as acute myeloid leukemia (AML), will require antigen-versatile targeting and off-switching of effectors in order to then allow rescue by hematopoietic stem cell transplantation (HSCT), preventing permanent myeloablation. To address this, we developed adaptor-CAR (AdFITC-CAR) T-cells targeting fluoresceinated AML antigen-binding diabody adaptors. This platform enables the use of adaptors matching the AML-antigen-expression profile and conditional activity modulation. Combining adaptors significantly improved lysis of AML cells in vitro. In therapeutic xenogeneic mouse models, AdFITC-CAR T-cells co-administered with single diabody adaptors were as efficient as direct CAR T-cells, and combinatorial use of adaptors further enhanced therapeutic efficacy against both, cell lines and primary AML. Collectively, this study provides proof-of-concept that AdFITC-CAR T-cells and combinations of adaptors can efficiently enhance immune-targeting of AML.

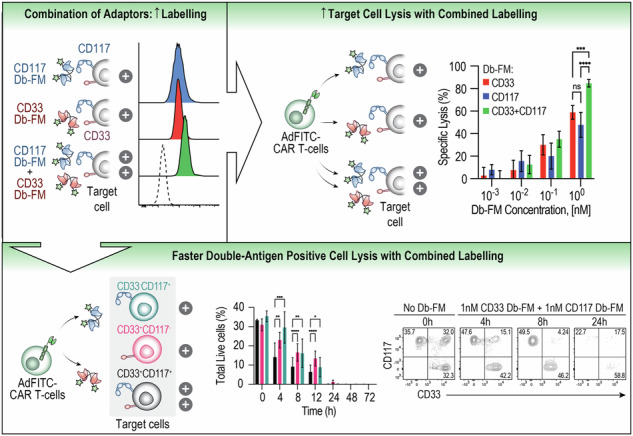

## Introduction

Chimeric antigen receptor (CAR) T-cells are T-lymphocytes, genetically engineered to display antigen recognition moieties on their surface, coupled to intracellular signaling modules that trigger T-cell activation upon antigen binding [1, 2]. Specifically, CAR T-cells directed against CD19 B-cell and BCMA plasma cell lineage antigens have led to improved clinical outcomes in patients suffering from advanced B-lymphoid and plasma cell malignancies [3–5]. These innovations represented paradigmatic first-in-class advances in cancer therapy [6]. However, several limitations of current CAR T-cell therapies are becoming evident: (a) low target antigen density might lead to insufficient CAR T-cell activity [7]; (b) therapeutic selection pressure might lead to outgrowth of antigen-negative tumor cells [8]; (c) single antigen-targeting may not generate a sufficiently high tumor-directed efficacy and selectivity; (d) production of currently applied patient-derived CAR T-cells results in fixed targeting modalities; (e) on-target off-tumor as well as off-target side-effects are difficult to control without inhibiting, and often definitively terminating CAR T-cells in the host [9]. Moreover, as the majority of targetable tumor antigens are not uniquely expressed on tumor cells but are also present on benign cellular counterparts (cell-of-origin), the on-target/off-tumor activity might pose a non-tolerable limitation in case the targeted cells, in contrast to e.g. the B-cell compartment, are critical for health and survival of the individual.

While (a–c) might be overcome by combinatorial tumor-antigen targeting, (d–e) might be addressed by flexible adaptor-mediated CAR T-cell (AdCAR T-cell) strategies. In these approaches, CAR T-cells recognize a specific tag and conditionally function in combination with tagged antigen-binding, tailored adaptors, which can be full antibodies, antibody fragments, or even small molecules, according to the desired affinity, tissue penetration capacity, and half-life [10]. The respective half-life of an adaptor allows for regulating CAR T-cell activity in an on-off manner, depending on clinical need and occurrence of side effects. Several AdCAR T-cell platforms have been described in preclinical studies [10, 11] and recently in a first clinical proof-of-concept setting [12]. Combinatorial use of bridging adaptors, enabling simultaneous targeting of multiple tumor-expressed antigens, could increase tumor specificity and tackle heterogeneous tumors, similarly to what has been achieved with dual CAR T-cells [13] and, more recently, with multi-target immunotherapy after multiplex editing of stem cells [14].

Acute Myeloid Leukemia (AML) and Myelodysplastic Neoplasia (MDS) develop from hematopoietic stem and progenitor cells (HSPCs) [15]. Despite high remission rates achieved with current combinatorial chemotherapeutic treatment regimens, including allogeneic HSPC and immune cell transplantation (mediating graft-versus-leukemia effects), the majority of patients experience relapse, which remains the principal cause of treatment failure and death [16, 17]. Therefore, more efficient tumor cell elimination remains the major therapeutic aim in these patients. CAR T-cells might serve this goal and are being assessed in early-phase clinical trials [18, 19]. However, the shared antigen expression on AML/MDS cells and on healthy HSPCs and more mature myeloid cells and the current limitation of knowledge on truly AML/MDS-restricted antigens pose a major challenge [20], as the continuous action of CAR T-cells would lead to permanent myeloablation [21]. We and others previously demonstrated that CAR T-cells could be used to abrogate both AML cells as well as HSPCs, and hematopoietic recovery would be ensured by HSPC transplantation after termination of CAR T-cell activity [22–24]. This approach could potentially be beneficial for patients unable to tolerate genotoxic preconditioning for HSPC transplantation.

We here hypothesized that AdCAR T-cells targeting AML cells *via* combinatorial use of bridging molecules, personalized for AML antigen-expression profiles, could efficiently eliminate tumor cells in a dose- and time-regulated manner. We thus generated and characterized AdCAR T-cells and two adaptor antibody constructs, targeting leukemic and cell-of-origin antigens CD33 and CD117. We show that the combinatorial use of adaptors increased the decoration of target cells and improved recognition and activation of CAR T-cells, leading to enhanced tumor cell lysis in vitro and in vivo.

## Methods

The studies involving primary human material were conducted in accordance with the Declaration of Helsinki and approved by the Cantonal Ethics Board Zürich, Switzerland (2009-0062). Healthy donors and AML patients gave written informed consent. Buffy coats from healthy donors were received with informed consent from the cantonal blood donation service, Zurich, Switzerland, and with ethical permission from the cantonal ethics committee Zurich. All procedures involving experimental animals were performed according to protocols approved by the Cantonal Veterinary Office Zurich in accordance with Swiss animal welfare laws and regulations under licenses 194/2019 and 134/2022. NSG (NOD.Cg-Prkdc^scid^ Il2rg^tm1WjI^/SzJ) mice were obtained from Charles River and bred in-house. Healthy male and female mice 6-9 weeks old were selected for in vivo experiments.

### Generation of AdFITC-CAR T-cells and direct CAR T-cells

The gene fragment encoding the scFv anti-fluorescein (E2) was cloned and inserted into the second-generation CAR lentiviral vector containing CD8α hinge-41BB-CD3ζ. The T2A-RQR8 gene sequence downstream of the receptor allowed the detection of the construct expression by staining the marker RQR8 with an anti-CD34 antibody (QBEND-10). The RQR8 gene sequence was kindly provided by Dr. Martin Pule (University College London, UK). CAR detection was performed by staining with AlexaFluor546-PEG_2_-FITC (AF546-FITC), generated as previously described [25]. Direct anti-CD33 (clone SGN-33) and CD117 (clone 79D) CAR T-cells were produced as reported from our lab [23, 24, 26] at 1500 × *g* for 1.5 h at 32 °C. Dynabeads were removed by magnetic separation 3 days after activation. CAR T-cells and control untransduced T-cells were expanded for a maximum of 12 days and then cryo-preserved.

### Protein production and conjugation

Sequences of the anti-CD33 (clone SGN-33) and CD117 (79D) variable domains were used as published in Co et al. [27] and Reshetnyak et al. [28], respectively. Diabodies were cloned with a 5-amino acid linker (GGSGG) between the variable domains into the mammalian expression vector pcDNA3.1(+) (Invitrogen, Thermo Fisher Scientific). An additional C-terminal cysteine was added after the hexahistidine tag for site-selective conjugations [29, 30]. Dbs were produced using transient gene expression (TGE) in CHO-S cells (Invitrogen) following standard protocols [31] and purified from the cell culture medium by immobilized nickel affinity chromatography using Ni-NTA (Roche). Site-selective conjugations of Db were performed by labeling engineered cysteine residues with fluorescein-5-maleimide (FM, Thermo Scientific) after TCEP-mediated reduction. In brief, antibody solutions in PBS (1 mg/ml) were reduced with 50 eq. of TCEP (Sigma Aldrich) in PBS overnight at 4°C. Following a washing step in Vivaspin™ columns (Sartorius), FM was dissolved in DMSO anhydrous to obtain a final concentration of 10% (v/v) when added to the reduced protein. Antibodies were treated with 20 eq. per cysteine of FM, and homogeneous conjugates with single modification were observed by LC-MS after 1 h. Buffer exchange of the conjugated proteins was performed through PD-10 Desalting Columns (Cytiva) in 50 mM Acetate Buffer (pH 5). Db-FM aliquots were snap-frozen in liquid nitrogen and stored at -80°C until further use.

### Primary human cells

All patient and healthy-donor cells were obtained from the biobank of the Department of Medical Oncology and Hematology, University Hospital Zürich, Zürich, Switzerland. Peripheral blood cells and bone marrow aspirates were acquired during initial diagnostics from patients. Patient characteristics of AML and myelodysplastic neoplasia (MDS) samples used for this study are indicated in Table S1. Cell collection and cryo-preservation procedures were performed as previously reported [32]. Residual, left-over healthy donor-derived bone marrow mononucleated cells (BM MNCs) and CD34-positively selected BM cells as well as G-CSF mobilized PB mononuclear cells were collected from stem cell infusion bags with permission of the Cantonal Ethics Committee of Zurich and cryopreserved until they were utilized for staining experiments. Mononuclear cells were enriched via density gradient centrifugation, and CD34+ HSPCs were isolated using FITC-labeled anti-CD34 antibodies (ThermoFisher) and anti-FITC microbeads with LS columns (Miltenyi Biotech), following the manufacturer’s guidelines. The negative and positive cell fractions were cryopreserved and stored in liquid nitrogen.

### Therapy studies in MOLM14 xenograft models

Leukemia engraftment was achieved by i.v. injection of 0.1×10^6^ MOLM14-CD117^high^ GFP^+^Luc^+^ cells after sub-lethal irradiation conditioning of mice (100 cGy; RS-2000 irradiator, Rad Source). Successful engraftment was confirmed at day 7 by BLI and mice were equally distributed according to flux signal to ensure uniformity among groups. Mice received i.v. administration of effector cells ranging from 10^7^ to 2 × 10^6^ cells. These included AdFITC-CAR T-cells, with or without concomitant Db-FM adaptor administration (twice daily, i.p.), or direct CAR T-cells. A negative control group comprised tumor-bearing mice that were i.v. injected with saline solution (PBS). Tumor burden was evaluated on a weekly basis by BLI. Except for day 7, images were set at the same luminescence scale. The experiments comparing the efficacy of direct CAR T-cells with the one mediated by AdFITC-CAR T-cells and Db-FM against the same antigen were terminated once the untreated arm reached predefined termination criteria. Combinatorial experiments were concluded after a 3-week course of antibody treatment. At terminal analysis, BM cells derived from one femur were resuspended in 1 ml single-cell suspension in FACS buffer and 200 μl analyzed by flow cytometry after the addition of counting beads (CountBright™ Absolute Counting Beads, Invitrogen). Contralateral femurs were kept in a solution of 4% paraformaldehyde (PFA, Santa Cruz Biotechnology) at 4°C until processing. Single-cell suspensions of 150 μl blood were obtained following incubation in red blood cell lysis buffer (Biolegend).

### Patient-derived AML xenografts and healthy human CD34+ cell transplantation and therapy in immunodeficient mice

Following sub-lethal irradiation of NSG mice, 5 × 10^6^ CD3^-^/CD19^-^ PB-derived primary AML patient cells (PID20) were intravenously injected. For therapy studies, seven days after engraftment, mice received either 10^7^ AdFITC-CAR T-cells, with or without concomitant administration of Db-FM adaptors (twice daily, i.p.), or 10^7^ direct anti-CD33 and CD117 CAR T-cells, administered alone or after pooling together 5×10^6^ monospecific CAR T-cells. At terminal analysis, flow cytometry analysis of BM, PB, and spleen was conducted to assess tumor burden. BM cells from one femur were resuspended in 1 ml of FACS buffer in a single-cell suspension, and 200 μl were analyzed by flow cytometry after the addition of counting beads. Single-cell suspensions of spleens (1 ml) and 150 μl of blood was obtained after incubation in red blood cell lysis buffer (Biolegend). For healthy human hematopoietic cell engraftment, sub-lethally irradiated NSG mice received 5×10^6^ CD34-selected M-PB HSPCs from an apheresis donor intravenously. Seven weeks post transplantation, human engraftment in peripheral blood was evaluated by flow cytometry. At week nine post-engraftment, 10^7^ AdFITC-CAR T-cells, generated from the same human donor, were injected i.v., with or without subsequent application of 12.5 μg CD117-Db FM i.p. every 12 hours for 10 days. After 10 days, mice were sacrificed and human chimerism was evaluated by flow cytometry. BM cells from one femur were resuspended in 1 ml of FACS buffer in a single-cell suspension, and 200 μl were analyzed by flow cytometry after the addition of counting beads.

## Results

### Generation of AdFITC-CAR T-cell and fluorescein-labeled diabody adaptor against AML target antigens CD117 and CD33

**Fig. 1:**
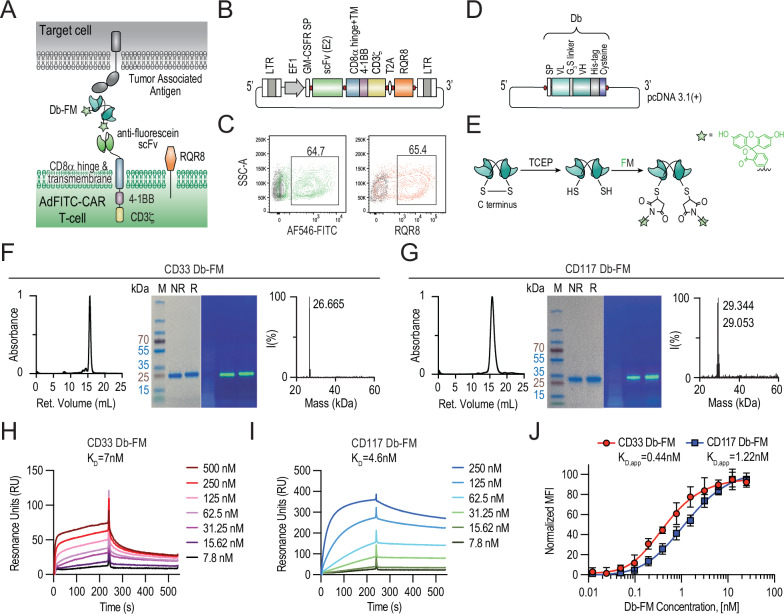
Generation of AdFITC-CAR T-cells and fluorescein-labeled antibody constructs. **A** Illustration of an AdFITC-CAR T-cell displaying anti-fluorescein scFv domain, targeting a generic tumor-associated antigen on a cell *via* a fluorescently labeled diabody adaptor as linking molecule. The surface co-expression of RQR8, which serves as a selection and depletion marker for AdFITC-CAR T-cells, is also depicted. **B** Schematic representation of second-generation CAR vector, presenting an anti-fluorescein scFv (clone E2). **C** Representative flow cytometry plots reflecting the transduction rate of healthy donor-derived T-cells are shown after staining with either AlexaFluor546-PEG_2_-FITC (AF546-FITC) in green or anti-CD34 antibody in red. Non-transduced T-cells served as control (black). **D** Plasmid map of the diabodies (Db) targeting CD33 and CD117 engineered to express a C-terminal cysteine. **E** Dbs were expressed and coupled with fluorescein-maleimide after a reduction step, as indicated in the protein conjugation scheme, yielding site-specifically labeled diabody (Db-FM). CD33 Db-FM (**F**) and CD117 Db-FM (**G**) were characterized by size exclusion chromatography (left), SDS-PAGE under non-reducing (NR) and reducing (R) conditions (middle; in addition, UV-light illuminated SDS-PAGE gels are also shown), and by mass spectrometry (right). M=Protein ladder indicating the kDa. Surface Plasmon Resonance analysis of CD33 Db-FM (**H**) and CD117 Db-FM (**I**) on a CM-5 chip coated with the extracellular domains of CD33 and CD117 His-tagged proteins, respectively. Dissociation constants K_D_ are indicated. **J** Binding activity of the CD33 Db-FM and CD117 Db-FM was determined by dose titration on MOLM14-CD117^high^GFP^+^Luc^+^ cells and detected by means of an APC-conjugated anti-FITC antibody. MFI was normalized to account for the higher level of CD117 antigen expression. Mean ± SD from triplicates. Resulting K_D,app_ are also indicated.

We generated AdFITC-CAR T-cells to display a single-chain variable fragment (scFv) binding fluorescein tagged to fluorescein-labeled diabodies, acting as bridging adaptors, leading to cross-linking and conditional activation of the CAR T-cell against a target cell, Fig. 1A. We cloned the anti-fluorescein CAR gene, based on the E2 antibody clone, in a second-generation lentiviral vector incorporating CD8α hinge and transmembrane domain followed by the intracellular portions of 4-1BB and CD3ζ chains [23, 24]. The vector also drives expression of the RQR8 marker [33], which can be recognized both by the clinical-stage anti-CD20 antibody rituximab for CAR T-cell depletion in vivo, and by anti-CD34 antibodies, allowing for CAR T-cell identification and immunomagnetic positive selection Fig. 1B. CAR T-cells were transduced as previously described [23] and transduction rates were confirmed via flow cytometry, as shown for representative samples in Fig. 1C.

Due to the high phenotype similarities between healthy and malignant HSPCs [34] and aiming to use the AdFITC-CAR T-cell diabody approach as both time-limited leukemia eliminating and non-genotoxic conditioning prior to subsequent HSPC-transplantation, we hypothesized that CD33 and CD117 could be highly suitable target antigens [35, 36]. We validated their cell surface expression by flow cytometry on AML cell lines and patient AML blasts, as well as on healthy-donor HSPCs and T-cells, alongside a panel of selected AML antigens, including CD38, CD123, and CD371 [37–39], Fig. S1. Both, AML cell lines (Fig. S1A) as well as primary human AML cells (Fig. S1B) exhibited varying levels of CD33 and CD117 expression, with primary human AML cells showing a somewhat higher degree of positivity for CD117 than cell lines. HSPCs isolated from four healthy donors were analyzed upon lineage exclusion and divided in more immature Lin^-^CD34^+^CD38^-^ and more mature Lin^-^CD34^+^CD38^+^ populations (Fig. S1C). While CD117 was similarly expressed in both populations, CD33 was up-regulated with HSPC maturation. Moreover, to gauge the risk of CAR T-cell fratricide, we analyzed the expression of these antigens on both stimulated and unstimulated T-cells (Fig. S1D). Apart from CD38, which was upregulated on stimulated T-cells, CD33 and CD117 expression was negative or comparatively low.

Based on these results and previously reported data [40–42], we confirmed and selected CD33 and CD117 for further AML targeting experiments and generated small antibody-based adaptors directed against CD33 and CD117. Considering that diabodies (Db) would be rapidly cleared from the body *via* the kidneys due to their molecular weight (~55 kDa), allowing for rapid control over AdFITC-CAR T-cell on-off activity on-demand, we cloned Db with C-terminal reactive cysteine residues [43, 44] (Fig. 1D). The respective antibody sequences are shown in Fig. S2. The diabodies were subsequently conjugated by site-specific labeling of the engineered cysteines with fluorescein-maleimide, leading to Db-FM conjugates, Fig. 1E. Db-FM adaptors were characterized by size-exclusion chromatography, SDS-PAGE with fluorescence detection, and mass spectrometry, confirming that constructs could be produced and fluorescently labeled, maintaining high levels of purity, Fig. 1F, G. Binding of the adaptors was confirmed by BIAcore analyses on chips coated with cognate antigens with dissociation constants in the low nanomolar range (Fig. 1H, I) and by staining MOLM14-CD117^high^GFP^+^Luc^+^ AML cells, positive for CD33 and CD117, exhibiting semi-saturation in the low nanomolar range (Fig. 1J). From this data, we conclude that this approach represents a viable strategy to generate AdFITC-CAR T-cells as well as highly homogeneous fluorescein-conjugated Db products with complete retention of target binding properties.

### AdFITC-CAR T-cells mediate tumor cell lysis dependent on the density of both target antigens and target-bound fluoresceinated adaptors

**Fig. 2:**
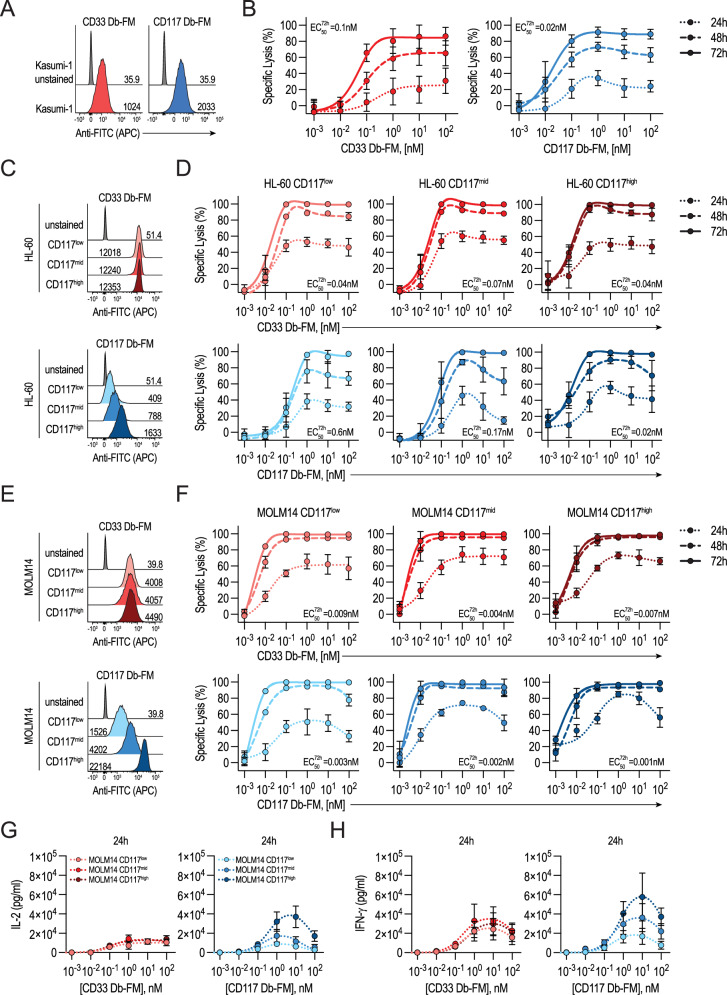
Db-FM adaptors elicit AdFITC-CAR T-cell cytotoxicity against various human AML cell lines in a concentration, time, and antigen density-dependent manner. **A** Representative histograms showing Kasumi-1 GFP^+^ cells stained with 10 nM CD33 Db-FM (left) and CD117 Db-FM (right), detected by APC-conjugated anti-FITC antibodies. MFI is indicated. **B** Kasumi-1 and AdFITC-CAR T-cells were co-cultured at an effector-to-target ratio E:T-1:1, tumor cell lysis was assessed via flow cytometry after 24, 48, and 72 h of co-culture. Percentage specific lysis of Kasumi-1 cells is shown relative to Db-FM concentration, represented as mean ± SD from three healthy-donor-derived AdFITC-CAR T-cell donors, each plated in duplicate wells. **C** Representative histograms showing HL-60 GFP^+^ cells transduced to express CD117 at high, medium, and low levels, labeled with 10 nM CD33 Db-FM (top) and CD117 Db-FM (bottom) as described in (**A**). **D** Percentage specific lysis of HL-60 cells was assessed over time after co-culture with AdFITC-CAR T-cells at E:T-1:1 and increasing levels of Db-FM. Mean ± SD from three healthy-donor AdFITC-CAR T-cell donors, each plated in duplicates. **E** Representative histograms showing MOLM14 GFP^+^ cells transduced to display various levels of CD117, stained and analyzed as described in (**C**). **F** MOLM14 cell lysis mediated by AdFITC-CAR T-cells at E:T-1:1 in culture with Db-FM. Percentage specific lysis of MOLM14 cells as a function of Db-FM concentration is represented as mean ± SD from three healthy-donor-derived AdFITC-CAR T-cell donors plated in duplicate wells. Quantification of IL-2 (**G**) and IFN-γ (**H**) in the supernatant from the same co-culture experiment indicated in (**F**) at 24 h.

We hypothesized that similarly to conventional direct CAR T-cells, also AdFITC-CAR T-cells would exhibit cytotoxic activity dependent on target antigen density [7, 23]. In our work, this effect might depend on the levels of target-antigen (CD33 and CD117) expression on the tumor cell surface, as well as on the density of the CAR T-cell ligand, fluorescein, decorating the tumor cell surface. To confirm expression levels of the target antigens on the three AML cell lines Kasumi-1, endogenously expressing CD33 and CD117, HL-60, and MOLM14, both expressing CD33 and transduced to display the extracellular domain of CD117 and sorted based on its expression level [23], we stained them with CD33 or CD117 Db-FM and detected the antigen-bound Db-FM with an APC-conjugated anti-FITC antibody (Fig. 2A, C, E). Next, we co-cultured AdFITC-CAR T-cells and titrations of both Db-FM in short-term in vitro experiments with Kasumi-1, HL-60, and MOLM14 cells, expressing various levels of CD117 target antigen (Fig. 2B, D, F). The resulting target cell lysis increased over time, depending on the concentration of adaptors, with EC_50_ in the sub-nanomolar range. As expected, CD117 antigen density affected AdFITC-CAR T-cell efficiency. Although the lysis of HL-60 CD117^high^ showed a 8-fold lower EC_50_ than HL-60 CD117^mid^ and 30-fold lower than HL-60 CD117^low^, a more moderate difference was observed in MOLM14 clones expressing various levels of CD117, where EC_50_ resulted to be 100-fold lower than HL-60 cells, regardless of the Db-FM. In line with this, the release of pro-inflammatory cytokines IL-2 and IFN-γ was positively correlated with the lytic activity of the CAR T-cells (Fig. 2G, H). Collectively, these data demonstrated that AML cell lysis and AdFITC-CAR T-cell activation were positively correlated with the amount of fluorescein-labeled Db adaptors and with antigen density on target cells.

### Combinatorial use of Db-FM adaptors increases biocidal activity of AdFITC-CAR T-cells against AML cell lines expressing the target antigens

**Fig. 3:**
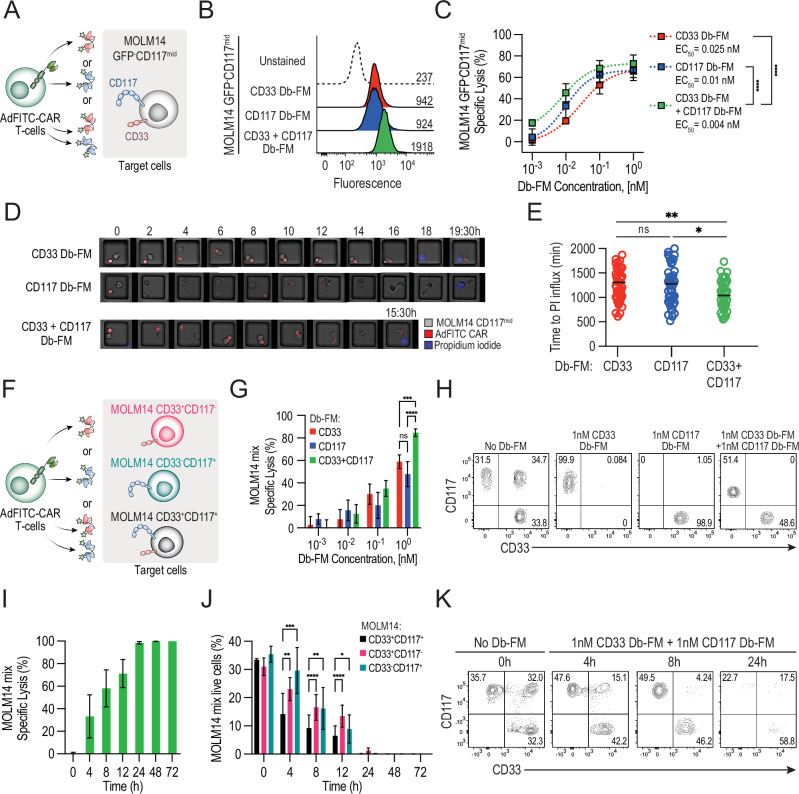
Combination of diabody adaptors enhances cytotoxicity against AML cell lines compared to single adaptors. **A** Experimental setup to evaluate adaptor-mediated AdFITC-CAR T-cell activity against MOLM14 cells, double positive for the targeted antigens CD33 and CD117. **B** MOLM14 GFP^-^CD117^mid^CD33^+^ cells were stained with 10 nM of either CD33 Db-FM, CD117 Db-FM, or both Db-FM (10 nM each). Unstained cells served as negative control. **C** Equal ratio of MOLM14-CD117^mid^CD33^+^ cells and AdFITC-CAR T-cells were cultured with increasing concentrations of CD33 and CD117 Db-FM, alone or in combination. Percentage specific lysis after 24 h is shown. Statistical analysis at the indicated EC_50_ was performed with one-way ANOVA, ***p < 0.001. **D**, **E** Time-lapse imaging to assess MOLM14-CD117^mid^CD33^+^ cell lysis by AdFITC-CAR T-cells in combination with CD33 and/or CD117 Db-FM. Time to PI influx was significantly shorter with the combinatorial adaptor approach when compared to single adaptor targeting (results from two healthy donors, statistical analysis conducted using one-way ANOVA; *p < 0.05; **p < 0.01; ***p < 0.001; ****p < 0.0001). **F** Experimental setup to evaluate adaptor-mediated AdFITC-CAR T-cell activity against MOLM14 cells, being either single or double positive for the targeted antigens. **G**, **H** MOLM14-CD117^neg^CD33^+^, MOLM14-CD117^high^CD33^+^, and MOLM14-CD117^high^CD33^KO^ cells were mixed at equal ratios and co-cultured with AdFITC-CAR T-cells at an E:T = 1:1. CD33 and CD117 Db-FM were added at 1 nM as single agents or in combination. **G** Percentage specific lysis of the target cells was measured at indicated time points. Statistical analysis was conducted using two-way ANOVA; ***p < 0.001; ****p < 0.0001. **H** Representative flow cytometry plots showing residual MOLM14 subpopulations at 24 h. **I**–**K** MOLM14-CD117^neg^CD33^+^, MOLM14-CD117^high^CD33^+^, and MOLM14-CD117^high^CD33^KO^ cells were mixed at equal ratios and co-cultured with healthy donor-derived AdFITC-CAR T-cells were mixed as in (**G**, **H**). The combination of CD33 and CD117 Db-FM was added at 1 nM, and target-cell lysis was measured at indicated time points. Percentage specific lysis of the overall target cells (**I**) and subpopulations (**J**) at the indicated time points. Statistical analysis was conducted using two-way ANOVA; *p < 0.05; **p < 0.01; ***p < 0.001; ****p < 0.0001. **K** Representative flow cytometry plots showing residual MOLM14 subpopulations at 0, 4, 8, and 24 h. **A**–**K** Unless otherwise indicated, data are presented as mean ± SD from three healthy donor-derived AdFITC-CAR T-cells, each plated in duplicate wells.

The observation that AdFITC-CAR T-cell biocidal activity was dependent on the adaptor concentration in our and previous results [7, 23] suggested that local increase of CAR-target (fluorescein) density, such as by simultaneous use of multiple tagged adaptors, could enhance the lytic efficacy. To test this hypothesis, we stained a cell line positive for both antigens, MOLM14-GFP^-^CD117^mid^, with saturating concentrations of CD117 and CD33 Db-FM as individual agents or in combination (Fig. 3A). As expected, we detected more than two-fold increased fluorescence upon combined staining (MFI = 924, 942 and 1918, respectively; Fig. 3B). Next, we interrogated whether increased fluorescein staining achieved by combinatorial use of multiple adaptors would also correlate to higher cytotoxic activity. Indeed, we observed enhanced tumor cell lysis when CD117 and CD33 Db-FM were added compared to equimolar concentrations of single agents alone (Fig. 3C). To further investigate the basis for increased lysis by dual targeting, we performed live cell imaging of effector-target cell interactions in microwells (Fig. 3D). The addition of both CD117 and CD33 Db-FM to the co-culture of MOLM14 CD117^mid^ and AdFITC-CAR T-cells resulted in significant faster tumor cell lysis (measured by influx of PI) as shown in Fig. 3E, which correlated with a more rapid, durable engagement time of CAR T-cells with target cells (Fig. S3A–E). Interestingly, no significant difference was observed in the time from initial durable engagement to lysis (Fig. S3D–E). Given the theoretical antigen-downregulation AML blasts could undergo during single-adaptor treatment, leading to escape of antigen-negative clones, we assessed the effect of the combination of adaptors on a heterogeneous tumor population. To test this, we titrated single Db-FM adaptors or equimolar concentrations of both adaptors on a pool of three transduced MOLM14 cell lines, engineered to express either CD117, or CD33, or both antigens (i.e. MOLM14-CD117^high^CD33^KO^, MOLM14-CD117^neg^CD33^high^ and MOLM14-CD117^high^CD33^high^) and recorded lytic activity mediated by AdFITC-CAR T-cells (Fig. 3F). The combinatorial use of 1 nM CD33 Db-FM and 1 nM CD117 Db-FM resulted in a significant increase in overall tumor cell lysis (Fig. 3G, H). Notably, despite the use of different cell lines in combination within the same culture wells, the lytic activity was primarily directed against cells expressing the cognate antigen of the specific adaptor utilized. Considering the correlation between enhanced cytotoxicity and increased surface-bound fluorescein, we hypothesized that combinatorial Db-FM treatment would lead to increased fluorescein staining of double-antigen-positive cells and enhanced lysis even in the presence of bystander single-antigen expressing cells. We thus co-cultured AdFITC-CAR T-cells in combination with CD33 and CD117 Db-FM against the same equal numbers of MOLM14 cells used in Fig. 3G, H and analyzed combined population lysis (Fig. 3I) alongside the sub-populations (Fig. 3J, K) over 72 h. At early time points after seeding (4, 8, 12 h), we observed significantly increased ablation of tumor cells expressing both CD33 and CD117 compared to only single-antigen-positive cells. At later time points, this effect was not detectable anymore when overall tumor cell lysis reached >90%.

These findings provide compelling evidence that a combination of adaptors targeting multiple tumor antigens contributes to enhanced AdFITC-CAR T-cell efficacy by increasing the fluorescein decoration of target cells, thereby overcoming limitations posed by the density of single antigen expression and possibly extending the targeting to heterogeneous antigen expressing tumors.

### Combination of Db-FM adaptors enhances AdFITC-CAR T-cells lysis of primary human AML blasts

**Fig. 4:**
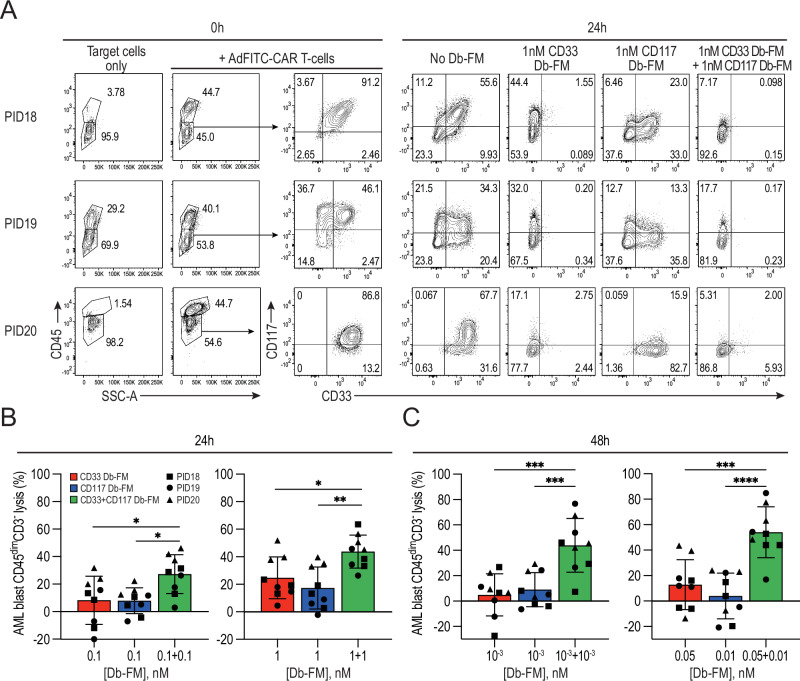
Combinatorial targeting of primary patient AML blasts is more efficient than single-adaptor targeting. **A** Surface expression of target antigens (CD33 and CD117) on CD45^dim^CD3^-^ cells from three AML patient samples. Representative flow cytometry analysis upon thawing, before and after the addition of healthy donor AdFITC-CAR T-cells at E:T = 1:1 ratio and after 24 h incubation in the presence of the indicated concentrations of diabody adaptors is shown. Percentage specific lysis of CD45^dim^CD3^-^ AML blast cells after 24 h (**B**) and 48 h (**C**) in culture with Db-FM targeting CD33 and CD117, alone or in combination. Data from 2 independent experiments with AML cells from three patients and AdFITC-CAR T-cells derived from 3 different healthy donors plated in duplicates (mean ± SD). Statistical analysis was conducted using two-way ANOVA; *p < 0.05; **p < 0.01; ***p < 0.001; ****p < 0.0001.

Given that the target antigens CD33 and CD117 are expressed with intra- and inter-patient heterogeneity on primary human AML blasts, we next investigated the efficacy of Db-FM and AdFITC-CAR T-cells against AML patient blasts in vitro. At thawing and after 24 h in culture with AdFITC-CAR T-cells, target cells exhibited a varied expression pattern of CD33 and CD117 (Fig. 4A). When effector and target cells were cultured together in the presence of defined concentrations of Db-FM, AdFITC-CAR T-cells were able to eliminate antigen-positive AML subpopulations, while sparing antigen-negative ones. The addition of both Db-FM, resulted in the lysis of the majority of blast cells, leading to a significantly improved cytotoxicity compared to equivalent concentrations of single Db-FM (Fig. 4A–C). This further supports the concept that combinatorial use of adaptors can substantially enhance the cytolytic efficacy of AdFITC-CAR T-cells, also against primary human AML blasts.

### In vitro and in vivo activation and cytokine release of AdFITC-CAR T-cells in absence of target-antigen expressing cells

Considering the observation that ligand-mediated dimerization can trigger CAR signaling even without the presence of antigen-positive cells [45, 46], we evaluated the induced AdFITC-CAR T-cell activation from crosslinking with bivalent Db-FM in absence of target-antigen expressing cells in vitro and in vivo. We incubated CAR T- and control T-cells, isolated from the same healthy donors and previously expanded in IL-2, in vitro for 72 h with or without addition of 10 nM CD117 Db-FM (Fig. S4A, B). We monitored their activation status at 24, 48 and 72 h by flow cytometry and compared to conditions where MOLM14-CD117^high^ target cells were added at an E:T = 1:1 to the co-culture (Fig. S4C, D). An upregulation of CD69^+^CD25^+^ was observed for RQR8^+^ AdFITC-CAR T-cells in culture with CD117 Db-FM with a progressive polarization towards CD69^-^CD25^+^ over time, especially visible in culture with target cells. The activation was accompanied by cellular clustering (Fig. S5A) and proliferation (Fig. S5B). In stark contrast however, significant levels of both IL-2 and IFN-γ were only released at high levels (10- and 280-fold increase, respectively) in presence of CD117-expressing MOLM14 cells (Fig. S5C). Importantly, neither Db-FM cultured with T-cells nor AdFITC-CAR T-cells without Db-FM resulted in T-cell activation, cytokine release and tumor cell lysis (Fig. S6A), indicating that biocidal activity was conditionally and selectively triggered by the concomitant presence of AdFITC-CAR T-cells and Db-FM.

We further evaluated AdFITC-CAR T-cell activation in vivo following multiple injections of CD117 Db-FM, which is not cross-reactive with murine CD117 [28]. NSG mice were injected i.v. with 10^7^ AdFITC-CAR T-cells followed by i.p administration of 25 μg Db-FM every 12 h, for a total of 4 doses over 48 h (Fig. S7A). Fluorescein was detectable on all AdFITC-CAR T-cells harvested from peripheral blood (PB), BM and spleen, and positively correlated with RQR8 expression (Fig. S7B). Importantly, the presence of Db-FM in vivo did not result in AdFITC-CAR T-cell activation nor T-cell expansion and did not lead to elevated levels of pro-inflammatory cytokines (Fig. S7C–F). Thus, although the presence of adaptors in absence of the target-antigen led to phenotypic AdFITC-CAR T-cell activation without cytokine production in vitro, this activation was not detectable in vivo when using biologically active Db-FM concentrations, suggesting that adaptor-mediated activation of AdFITC-CAR T-cells in the absence of target antigen likely will not pose a relevant toxicity in vivo.

### Db-FM adaptors exhibit short in vivo serum half-lives while maintaining longer on-tumor residence time

**Fig. 5:**
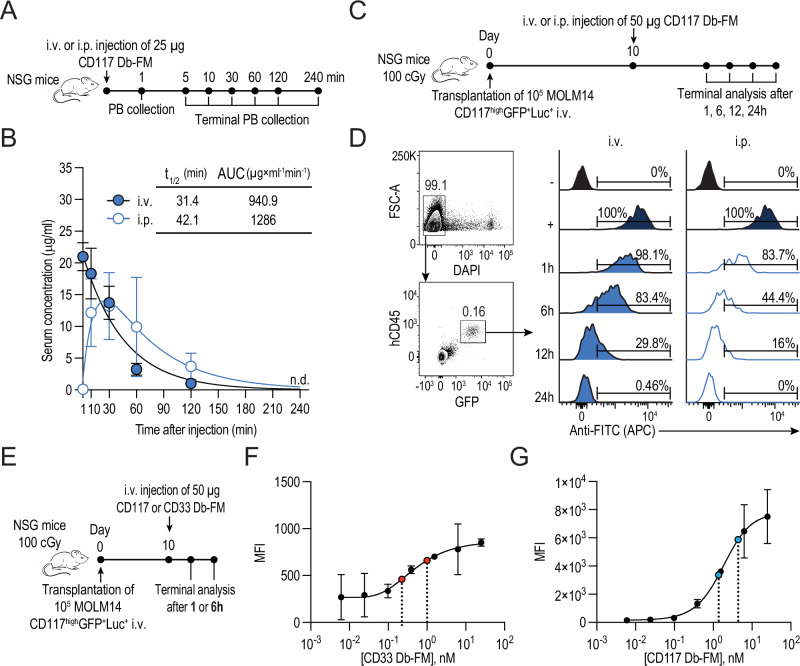
In vivo pharmacokinetic (PK) and pharmacodynamic (PD) properties of Db-FM adaptors. **A** Schematic representation of PK experimental set-up in mice not carrying target cells. NSG mice received a single i.v. or i.p. injection of 25 μg CD117 Db-FM (*n* = 2 mice per time point per administration route). Blood was collected from all mice at 1 min post-injection and terminally at the indicated time points. **B** Db-FM concentration in serum was determined by ELISA (mean ± SD of technical duplicates per mouse). Calculated t_1/2_ and AUC are indicated. **C** Experimental set-up to study on-tumor residence time of CD117 Db-FM on MOLM14-CD117^high^ GFP^+^Luc^+^ cells in xenografted mice. NSG mice were sublethally irradiated and injected i.v. with 10^5^ MOLM14 cells. On day 10, 50 μg of CD117 Db-FM were injected i.v. or i.p. (*n* = 1 mouse per time point per administration route). **D** At the indicated time points, live MOLM14 cells (hCD45^+^GFP^+^) isolated from BM were analyzed by flow cytometry (representative dot plot on the left), and target-bound CD117 Db-FM was detected by APC-conjugated anti-FITC antibody staining (histograms on the right). MOLM14 cells from mice engrafted but not subsequently injected with Db-FM served as negative (−) and positive controls after ex vivo staining with 10 nM CD117 Db-FM (+). **E** Sublethally irradiated NSG mice were injected i.v. with 10^5^ MOLM14-CD117^high^GFP^+^Luc^+^ cells. On day 10, 50 μg of CD117 or CD33 Db-FM were injected i.v. and femora were collected after 1 and 6 h to determine the clearance of Db-FM from the tumor cell surface by flow cytometry (*n* = 1 mouse per time point per Db-FM). A mouse engrafted with MOLM14 but not injected with adaptors served as negative control. **F**, **G** After ex vivo staining of cells isolated from the control mouse with titration of Db-FM, the surface labeling levels obtained from known Db-FM concentrations were used to interpolate the concentrations of Db-FM on tumor cells retrieved from mice injected with Db-FM. MFI resulting from target-bound Db-FM was detected by APC-conjugated anti-FITC antibody. **F** Interpolated on-tumor CD33 Db-FM concentration was estimated at 0.99 nM after 1 h injection and 0.22 nM after 6 h (each indicated in red). **G** Interpolated on-tumor CD117 Db-FM concentration was determined to be 4.37 nM after 1 h injection and 1.37 nM after 6 h (each indicated in blue).

To study the in vivo half-life of Db-FM adaptors, we administered 25 μg of CD117 Db-FM either intravenously (i.v.) or intraperitoneally (i.p.) to NSG mice and collected blood 1 min post-injection and terminally at the indicated time points (Fig. 5A). Serum half-life accounted for approximately 30 minutes upon i.v. injection and 40 minutes for i.p. injection, with possibly slightly longer exposure to Db-FM following i.p. injections, as indicated by the values of area under the plasma concentration-time curve (AUC) in Fig. 5B. Before studying the residence time of Db-FM:tumor complex in vivo, we assessed the growth kinetics of MOLM14 cells in NSG mice at two different doses (1 and 2.5 × 10^5^) by correlating bioluminescence flux with terminal flow cytometry analysis and immunohistochemistry staining (Fig. S8A–E). Remarkably, flow analysis of BM underestimated MOLM14 engraftment levels when compared to histology analysis (Fig. S8E). Based on our data, injection of 1 × 10^5^ consistently resulted in detectable bioluminescence signals by day 7, with formation of tumor cell infiltrates in the bone marrow (BM), confirmed by HE and CD117 immunohistochemistry staining. To determine the in vivo residence time of the Db-FM adaptor on tumor cells, we injected 50 μg of CD117 Db-FM in NSG mice, engrafted ten days prior with 1 × 10^5^ MOLM14-CD117^high^ GFP^+^Luc^+^ cells (Fig. 5C). Db-FM was detectable on MOLM14 cells in decreasing intensity for up to 12 h after i.v. or i.p. injection by flow cytometry (Fig. 5D). This decrease of in vivo labeling over time is likely due to the concomitant effects of the Db-FM off-rate and fast Db-FM clearance as well as the exponential growth of MOLM14 cells in vivo and therefore dilution of surface-bound Db-FM (Fig. S8C, D).

We next evaluated the in vivo tumor-residence time of both adaptors by injecting tumor-bearing mice with CD33 or CD117 Db-FM (Fig. 5E). The intensity of MOLM14 cells retrieved from mice injected with Db-FM 1 and 6 h prior to terminal analysis, was compared to the staining from ex vivo titrations of CD33 or CD117 Db-FM (Fig. 5F, G). Assuming an average plasma volume of 49 μl/g [47] and an immediate distribution in the blood compartment, the theoretical initial concentration of Db-FM in serum accounted on average for 744 nM. Based on the interpolated concentration of tumor-bound Db-FM (Fig. 5F, G), CD117 Db-FM displayed higher MFIs (consistent with the antigen expression levels), with on-tumor concentrations starting at 4.4 nM 1 h after injection, with a 3-fold decrease observed between 1 and 6 h post-injection. Conversely, CD33 Db-FM presented a relatively lower density on the tumor cell surface (1 nM) and experienced a 4.5-fold reduction in surface labeling over the same time, possibly explained by receptor-mediated internalization of CD33 Db-FM [48].

Based on the determined on-tumor residence time of Db-FM, we designed a dosing regimen with i.p. Db-FM injections scheduled at 12 h intervals, aiming to maintain sufficient fluorescein density on MOLM14 cell surface and counteract the rapid clearance from the tumor cell surface during the in vivo therapy phase.

### AdFITC-CAR T-cells in combination with CD33 or CD117 Db-FM deplete MOLM14 cells as efficiently as direct antigen-targeting CAR T-cells in vivo

**Fig. 6:**
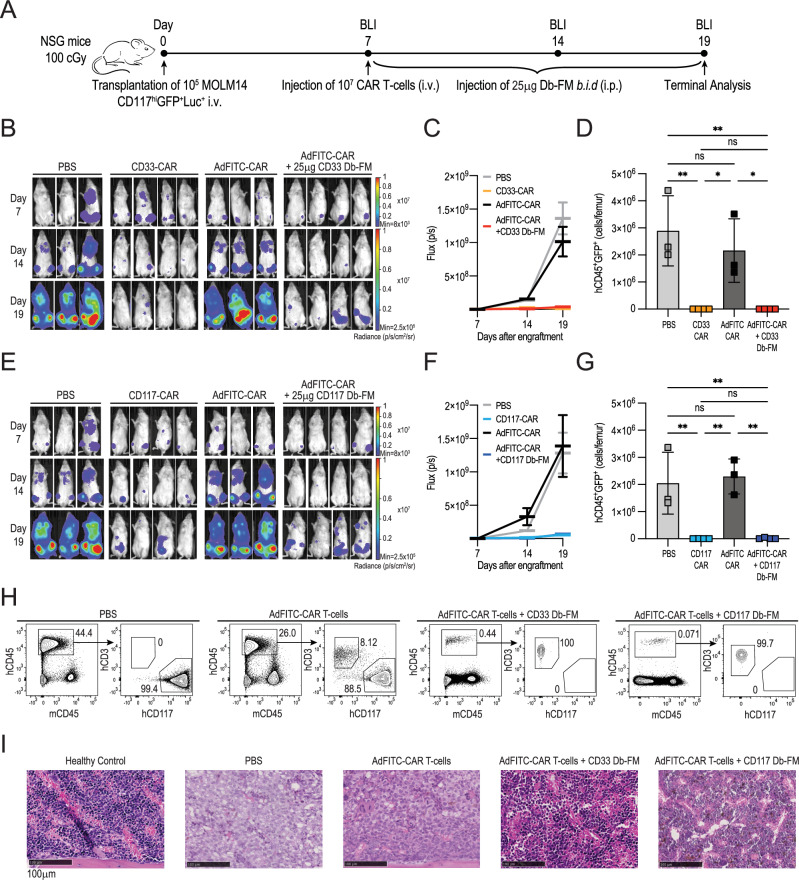
In vivo therapeutic efficiency of direct CAR T-cells and AdFITC-CAR T-cells in combination with CD33 or CD117 Db-FM adaptors against MOLM14-CD117^high^AML cells. **A** Schematic outline of the experimental setup. Sub-lethally irradiated mice were engrafted with 10^5^ MOLM14-CD117^high^GFP^+^Luc^+^ cells. Seven days later, engraftment was confirmed by bioluminescence analysis (BLI), and mice were injected with either 10^7^ direct CAR T-cells targeting CD33 or CD117 (positive controls) or AdFITC-CAR T-cells (*n* = 3/4 mice per group). Mice from the latter group subsequently received 25 μg CD33 Db-FM or CD117 Db-FM (i.p. every 12 h) or no adaptors (negative controls). **B** Bioluminescence analysis of MOLM14 cell engraftment at days 7, 14, and 19 in mice treated with PBS, AdFITC-CAR T-cells in the absence or presence of adaptor, or direct CAR T-cells (CD33-CAR). **C** Quantification of the bioluminescence flux of whole-body imaging of mice in (**B**). **D** Absolute counts of hCD45^+^GFP^+^ MOLM14 cells (mean ± SD) in the BM of a single femur per mouse at terminal analysis. Statistical analysis was conducted using two-way ANOVA; *p < 0.05, **p < 0.01. **E** Bioluminescence analysis at indicated time points of mice treated with PBS, AdFITC-CAR T-cells with or without adaptor, or direct CAR T-cells (CD117-CAR). **F** Corresponding quantification of the bioluminescence flux of mice showed in (**E**). **G** Absolute hCD45^+^GFP^+^ MOLM14 cell count (mean ± SD) in the BM of a single femur per mouse at the end of the study, statistical analysis performed as indicated in (**D**). **B**–**G** Data shown from two independent experiments. **H** Representative flow cytometry plots of live cells from BM single-cell suspensions receiving the above-indicated treatment. **I** HE staining of the contralateral femora from the mice depicted in (**H**), as well as from healthy age-matched control mice (20x magnification, solid line indicates 100 μm).

We next tested AdFITC-CAR T-cell cytotoxic activity in vivo. Following confirmation of MOLM14-CD117^high^GFP^+^Luc^+^ engraftment at day 7 by bioluminescence imaging of NSG mice, we administered i.v. either 10^7^ direct anti-CD33 or anti-CD117 CAR T-cells, or 10^7^ AdFITC-CAR T-cells followed by 25 μg CD33 or CD117 Db-FM i.p. every 12 h, respectively (Fig. 6A). At day 19, mice were sacrificed as untreated control mice met termination criteria. Bioluminescence imaging showed inhibition of tumor growth in mice treated with direct CAR T-cells and AdFITC-CAR T-cells in combination with the respective adaptors (Fig. 6B, C, E, F). At terminal analysis, we observed successful eradication of MOLM14 cells in the BM of treated mice by flow cytometry and reconstitution of visually relatively normal marrow by histology analysis (Fig. 6D, G–I). Some mice exhibited low-level extra-medullary engraftment of MOLM14 cells, as detected by bioluminescence imaging, in both the direct and adaptor-mediated CAR T-cell experimental groups. Importantly, deviation from the 12 h administration schedule, such as administering antibody every 48 h, abolished the therapeutic effect (Fig. S9A–C), indicating that maintaining continuous and adequate coverage of adaptors on the surface of tumor cells is crucial for therapeutic success. These data collectively underscore the comparable efficacy of AdFITC-CAR T-cells in combination with their respective adaptors and direct CAR T-cells in xenogeneic therapy studies. Moreover, considering the tumor engraftment in presence of AdFITC-CAR T-cells but absence of adaptors, this suggests that the system would allow for off-control of AdFITC-CAR T-cell function in vivo.

We next evaluated the potency of AdFITC-CAR T-cells by limiting their dose in vivo, starting from 10^7^ and decreasing fivefold down to 0.2 × 10^7^. A reduction in CAR T-cell numbers resulted in a reduced therapeutic response, as assessed by bioluminescence imaging, with a more pronounced effect in the context of adaptor-CAR T-cell approaches compared to direct CAR T-cells (Fig. S10A–D). These findings highlight the importance of establishing a sufficient effector-to-target ratio at start of therapy. Considering the estimated doubling time ranging here from 0.6 and 0.9 days, as determined from tumor growth kinetics based on bioluminescence signal and flow cytometry (Fig. S8C, D), it can be estimated that at least 9 × 10^6^ MOLM14 target cells would be present in mice at the start of the therapy, leading to an E:T of about 1:1 when 10^7^ CAR T-cells are transferred. In sum, these findings underline that the effectiveness of AdFITC-CAR T-cells in our exemplary model system is, as expected, dependent on both the dosage of CAR T-cells and a regimen enabling their sustained activation in vivo.

### Combinatorial use of adaptors leads to most efficient AdFITC-CAR T-cell mediated AML cell line elimination in a therapeutic setting in vivo

**Fig. 7:**
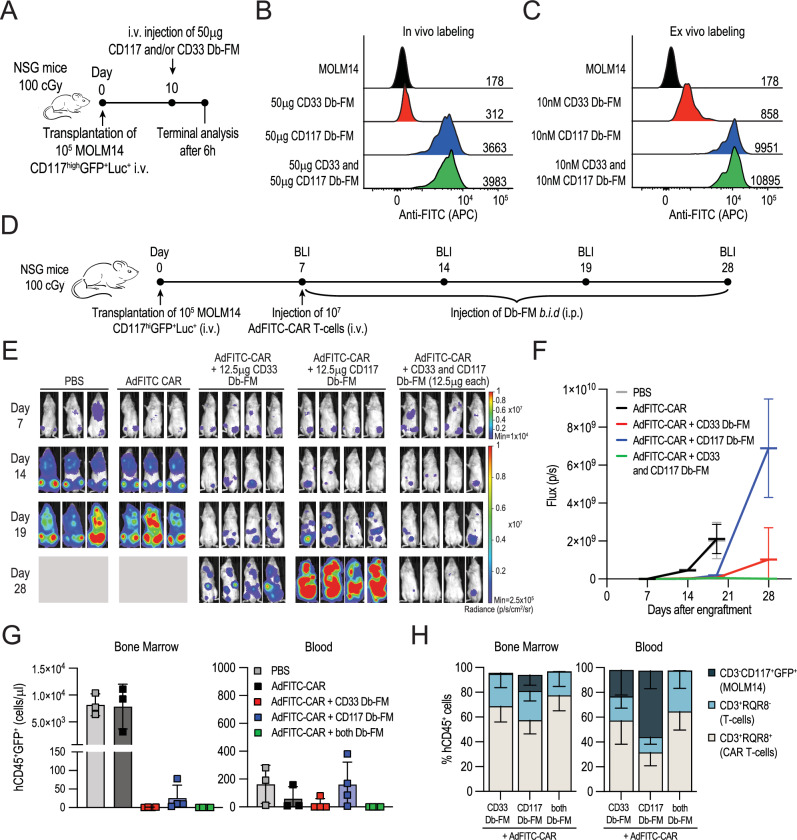
Combinatorial use of adaptors improved in vivo therapeutic efficacy of AdFITC-CAR T-cells against MOLM14-CD117^high^ AML cells. **A** Sublethally irradiated NSG mice were i.v. injected with 10^5^ MOLM14-CD117^high^GFP^+^Luc^+^ cells. On day 10, 50μg of CD117 or CD33 Db-FM were administered i.v. alone or in combination, and BM was collected 6 h post injection (*n* = 1 mouse per condition). **B** Flow cytometry histograms showing APC signal from anti-fluorescein antibodies targeting the Db-FM bound to MOLM14 cells at terminal analysis. MFIs indicated on the histograms show increased staining, obtained by combinatorial administration of adaptors. **C** MOLM14 cells from engrafted mice not receiving Db-FM injection were used as negative control and labeled ex vivo with 10 nM Db-FM alone or in combination to compare with in vivo labeling in (**B**). MFI is reported on the histograms. **D** Schematic experimental setup. NSG mice were sublethally irradiated and engrafted with 10^5^ MOLM14-CD117^high^GFP^+^Luc^+^ cells. After seven days, mice were injected with 10^7^ AdFITC-CAR T-cells i.v. and subsequently with 12.5 μg CD33 and CD117 Db-FM i.p. alone or in combination every 12 h for three consecutive weeks (*n* = 3/4 mice per group). **E** In vivo tumor burden evaluation by bioluminescence imaging at indicated time points. On day 28, placeholders indicate mice previously terminated due to high tumor load. **F** Quantification of BLI flux signal over time (mean ± SD). **G** Absolute counts of hCD45^+^GFP^+^ MOLM14 cells (mean ± SD) isolated from BM of one femur each and from 150 μl of blood at terminal analysis. Counts refer to a final resuspension volume of 1 ml. **H** Percentages of MOLM14 cells, T-cells, and AdFITC-CAR T-cell fraction within total hCD45^+^ cells (mean ± SD) in BM and blood, identified based on surface expression of CD3, CD117, and RQR8 by flow cytometry.

Building on these findings, we sought to evaluate the therapeutic efficacy of combinations of adaptors in vivo against an AML cell line. First, we investigated whether simultaneous administration of both adaptors would also result in increased fluorescein intensity on tumor cells isolated from the BM at terminal analysis, similarly to what we already observed in vitro (Fig. 3A). NSG mice were engrafted with MOLM14-CD117^high^GFP^+^Luc^+^ cells, and 10 days later, injected i.v. with 50 μg of CD33 and CD117 Db-FM alone or in combination (Fig. 7A). Indeed, concurrent administration of both adaptors resulted in the highest MFI (Fig. 7B), recapitulating the delta in intensity observed after ex vivo staining of cells isolated from a mouse not receiving Db-FM (Fig. 7C). Next, we treated tumor-bearing mice with AdFITC-CAR T-cells and either 12.5 μg CD117 or CD33 Db-FM as monotherapies or a combination of both adaptors for three weeks starting from seven days after MOLM14-CD117^high^GFP^+^Luc^+^ injection (Fig. 7D). At day 28, mice treated exclusively with CD33 and CD117 Db-FM experienced the occurrence of extramedullary manifestations as myeloid sarcomas. Conversely, in mice treated with a combination of Db-FM, tumor growth was effectively inhibited as indicated by a lower overall bioluminescence signal (Fig. 7E, F). Flow cytometry analysis at the end of the experiment indicated that AdFITC-CAR T-cells with adaptors (alone or in combination) reduced the tumor engraftment in BM and blood (Fig. 7G) accompanied by expansion and persistence of AdFITC-CAR T-cells after infusion (Fig. 7H). Together, these data demonstrate that combinatorial targeting of antigens with adaptor-based CAR T-cell strategies leads to more efficient AML cell elimination in vivo.

### Combinatorial use of adaptors leads to enhanced AdFITC-CAR T-cell mediated primary AML xenograft elimination in a therapeutic in vivo setting

**Fig. 8:**
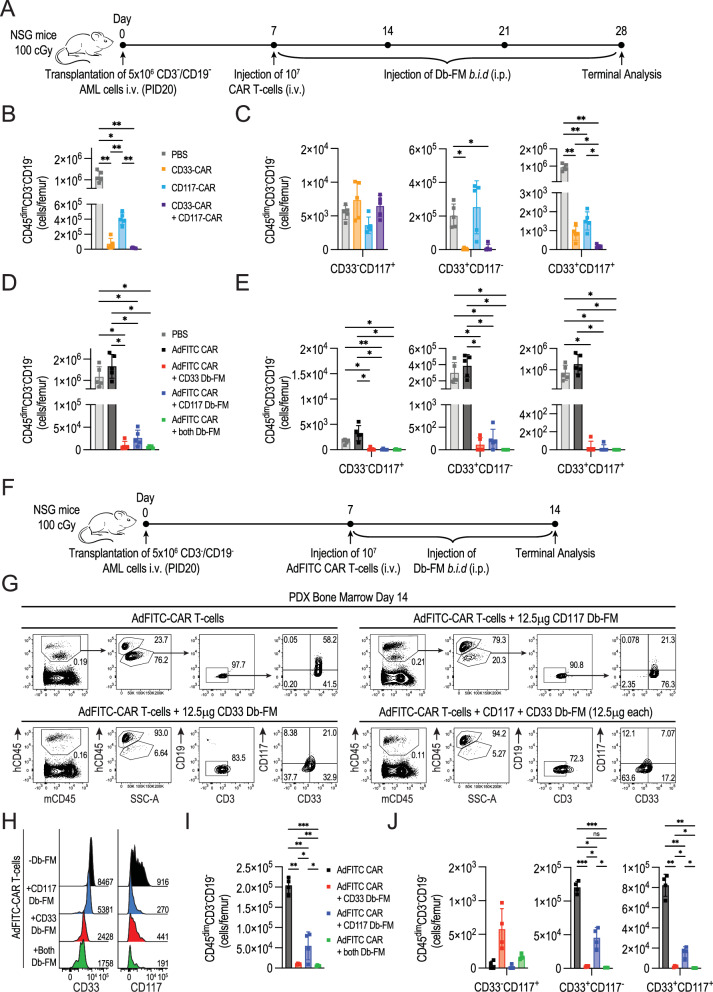
Combinatorial use of adaptors enhances the therapeutic efficacy of AdFITC-CAR T-cells against primary patient AML cells in murine xenograft models. **A** Schematic experimental setup. Seven days after engraftment with 5 × 10^6^ CD3/CD19 double-depleted PB cells isolated from AML patient PID20, sublethally irradiated NSG mice were injected i.v. with either 10^7^ direct anti-CD117 or direct anti-CD33 CAR T-cells (alone or 5×10^6^ each in combination), or 10^7^ AdFITC-CAR T-cells. Subsequently, 12.5 μg CD33 and/or CD117 Db-FM were injected i.p. every 12 h for three weeks (*n* = 5 mice per group). **B**–**E** Absolute counts of residual AML cells (hCD45^dim^CD3^-^CD19^-^) and respective subpopulations expressing the target antigens CD33 and CD117 (mean ± SD) in the BM of a single femur per mouse at terminal analysis. Statistical analysis was conducted using one-way ANOVA; *p < 0.05, **p < 0.01. **F** Schematic experimental setup. Seven days after engraftment with 5×10^6^ PID20 AML cells, mice were injected with either 10^7^ AdFITC-CAR T-cells i.v. followed by i.p. administration of 12.5 μg CD33 and CD117 Db-FM, alone or in combination, at 12 h intervals for one week (*n* = 4 mice per group). **G** Representative flow cytometry plots of live cells from BM single-cell suspensions at terminal analysis for each experimental group. **H** Representative flow cytometry histograms showing the target antigen levels in mice receiving the indicated treatments. MFIs are reported on the histograms. **I**, **J** Absolute counts of residual AML cells (hCD45^dim^CD3^-^CD19^-^) and respective subpopulations expressing CD33 and CD117 (mean ± SD) in the BM of a single femur per mouse. Statistical analysis was conducted using one-way ANOVA; *p < 0.05, **p < 0.01, ***p < 0.001.

Expanding on the beneficial tumor control observed with combined adaptor administration, we assessed the therapeutic effectiveness of adaptor combinations in treating mice engrafted with primary AML patient samples (Fig. 8A). We examined primary AML (PID20) engraftment kinetics after intravenous administration of 5 × 10^6^ CD3/CD19 double-depleted PB cells in NSG mice through flow cytometry and immunohistochemistry analysis of the hematopoietic organ chimerism over the course of seven weeks. We observed a rapid disease onset with detectable blasts in blood, bone marrow and spleen starting from day 7 after engraftment until bone marrow cell necrosis at day 49 (Fig. S11A–D). Immunophenotyping of engrafted CD45^dim^CD3^−^CD19^−^ cells showed CD33 and CD117 expression, consistent with the staining pattern of AML cells upon thawing (Fig. 4A). Next, we investigated if direct anti-CD33-CAR or anti-CD117-CAR T-cells could effectively target primary AML cells. Seven days after transplantation of 5 × 10^6^ primary AML cells, mice were treated with 10^7^ direct CAR T-cells. Flow cytometry analysis performed three weeks after CAR T-cell injection showed reduction of the majority of infiltrating BM blasts in all treated conditions (Figs. 8B and  S12A). Upon combination of both 5 × 10^6^ anti-CD33 and anti-CD117 CAR T-cells, we observed a significant reduction of the CD33^+^CD117^+^ blast subpopulation, while antigen-negative cells evaded targeting when single direct-CAR were administered (Fig. 8C).

Based on these data, we subsequently investigated whether a similar therapeutic outcome could be achieved by administering 10^7^ AdFITC-CAR T-cells and Db-FM at a 12.5 μg dose every 12 h for three weeks starting from day 7 after tumor engraftment. While AdFITC-CAR T-cells in combination with both Db-FM exhibited the highest tumor eradication at terminal analysis, mice treated with single Db-FM demonstrated comparable effectiveness, successfully eliminating the majority of tumor cell infiltrates in the BM (Figs. 8D, E and S12A). Notably, the administration of healthy-donor derived AdFITC-CAR T-cells in absence of bridging adaptors was unable to control tumor growth in the PDX model. Considering the high efficacy of AdFITC CAR-T cells against AML blasts in this setting, regardless of the Db-FM administered, we reasoned that terminating the experiment after one week of treatment could elucidate the varying kinetics of AML eradication, following administration of adaptors as single agents or in combination (Fig. 8F). At day 14, we analyzed the immunophenotype of residual CD45^dim^CD3^-^CD19^-^ AML blasts by flow cytometry (Fig. 8G). Leukemic cells isolated from mice treated with AdFITC-CAR T-cells and Db-FM showed a decreased MFI with respect to the targeted antigens compared to those receiving only AdFITC-CAR T-cells, with the most substantial reduction when both adaptors were administered simultaneously (Fig. 8H). Although AdFITC-CAR T-cells and single Db-FM significantly reduced tumor cell infiltration in the BM (Fig. 8I), the combination of Db-FM proved to be the most effective in reducing AML cells and the most efficient in targeting the double-positive CD33^+^CD117^+^ subpopulation (Fig. 8I, J). This is consistent with what we previously observed in vivo using the MOLM14 cell line, positive for both targeted antigens. Taken together, this data shows that combinatorial targeting of different antigens *via* adaptor-based CAR T-cells is most efficient in an in vivo therapeutic model system with primary human cells, suggesting that this approach might represent a versatile strategy to overcome limitations of low and heterogenous antigen expression in therapeutic settings of AML treatment.

### AdFITC-CAR T-cells in combination with CD117 Db-FM deplete healthy human CD117-positive cells in xenografted mice

To evaluate the on-target toxicity of AdFITC-CAR T-cells targeting CD117 *via* CD117 Db-FM, we transplanted NSG mice with mobilized peripheral blood (M-PB) CD34^+^ cells from a healthy donor. Upon confirming human multilineage hematopoietic entraftment in PB, we administered AdFITC-CAR T-cells and subsequently applied 12.5 μg CD117 Db-FM twice daily for 10 days before terminal analysis of mice (Fig. S13A, B). While control mice maintained CD117-expressing human cells in the bone marrow, these cells were substantially reduced (over 90%) in mice treated with AdFITC-CAR T-cell and CD117 Db-FM, as shown for CD34^+^CD38^-^ and CD34^+^CD38^+^ human HSPCs sub-populations (Fig. S13C, D). Although mCD45^+^ counts were similar across all conditions, overall hCD45^+^ engraftment decreased as an effect of AdFITC-CAR T-cell plus CD117 Db-FM treatment, likely due to prolonged targeting of the apex of the hematopoietic hierarchy (Fig. S13E). Interestingly, also human mast cells, expressing highest CD117 levels, were significantly reduced in AdFITC-CAR T-cell plus CD117 Db-FM treated animals (Fig. S13C, E). These findings indicate the therapeutic efficacy of AdFITC-CAR T-cells in combination with CD117 Db-FM against healthy CD117-expressing cells in vivo, supporting the potential use of this approach to not only target AML cells, but also to reduce in a time-dependent manner healthy HSPCs as a pre-conditioning regimen prior to healthy HSPC transplantation.

## Discussion

Focusing on AML as a paradigmatic hematopoietic stem and progenitor cell disease with high clinical need for therapeutic improvement [16, 49], we here addressed several challenges of current CAR T-cell therapies [7, 9, 50]. We show that a) adaptor-mediated tumor-cell lysis *via* AdCAR T-cells can be as efficient as direct targeting CAR T-cells, which provides a basis for using a single CAR construct in combination with adaptors, aligned to individual tumor-antigen expression, and that b) combined use of adaptors, expressing the same CAR-activation tag, but targeting two different tumor-associated antigens, enhance CAR T-cell biocidal activity. The latter effect can be ascribed to both, increased CAR T-cell recognized antigen density (with respect to double target-antigen expressing cells) as well as to more efficient elimination of primary, heterogeneous antigen expressing cells, i.e. antigen-diversified tumor cell populations.

Our findings align with prior studies, demonstrating a positive correlation between antigen expression and CAR T-cell biocidal activity [7, 23]. In addition, the challenge of low expression of target antigens, which hinder sufficient CAR T-cell activation, could be overcome by combinations of adaptors carrying the same CAR T-cell recognition domain (e.g. fluorescein), potentially enabling the activation threshold necessary for CAR T-cell cytotoxicity. Given that most targetable, MHC and non-MHC presented antigens are not “tumor-private” antigens but are shared with non-malignant counterparts, i.e. the cells-of-origin, differential antigen density might be exploited to increase tumor-cell-directed specificity [51]. Here, we show that combining two tumor-targeting adaptors resulted in significantly higher tumor cell death in both AML cell lines and primary patient AML cells in vitro and in vivo, in line with recently published combinatorial in vitro targeting studies on leukemia cell lines [52].

As AML arises from HSPCs via subsequent genetic events [53], few AML-private antigens, distinct from HSPCs and suitable for immune-targeting, have been identified [40–42]. Complementing previous studies [13, 40, 41], we observed shared antigen expression with HSPCs and highly heterogenous expression on both AML cell lines and primary AML cells, making a combinatorial targeting approach a potentially suitable strategy, which might achieve effective AML elimination but will also lead to the ablation of healthy HSPCs. The time-limited on-off AdCAR T-cell combinatorial adaptor approach could then potentially serve as an HSPC transplantation pre-conditioning approach, obviating the use of cytotoxic conditioning [23, 24, 54–56]. In addition, although not directly proven in this study, it could be speculated that combinatorial use of adaptors could improve the safety profile of CAR T-cells by mitigating the on-target collateral damage to non-HSPC healthy tissues, expressing one antigen of the combination or both antigens at lower levels, similar to what has been shown with the employment of IF-BETTER and fine-tuned AND logic gating strategies for CAR T-cell design [13, 57].

The molecular weights of the adaptor molecules determine their plasma half-life and tissue penetration, while factors like target-affinity, target-mediated internalization and tumor growth, and thus antigen-dilution, contribute to the on-target residence time of the adaptor molecule. In addition, adaptor size, as well as the specific epitope proximity to the target cell membrane might influence biocidal activity of the AdCAR T-cell [58]. While Db-FM presented serum half-lives of approximately 30-40 minutes, the on-tumor residence time varied, depending on tumor-cell doubling, target-antigen affinity and antigen density. These findings indicate that effective dosing of individual adaptors might need to be specifically tailored, and different treatment strategies, including intermittent adaptor dosing regimens to possibly further enhance AdCAR T-cell efficacy [48, 59], should be investigated. Nevertheless, the on-target residence time of the adaptors used in our study of less than twelve hours should allow HSCT within a time window of a few days after adaptor termination.

Several AdCAR T-cells specific for various tags, and multiple tagged adaptors are being developed in both preclinical and clinical settings [12, 48, 59, 60]. We chose fluorescein as CAR ligand as it is approved as an in vivo diagnostic dye [61] without raising safety concerns [60], and fluorescein can be readily conjugated to a plethora of target-binders [10, 62–64], including already clinically approved antigen-binding molecules [52, 60]. While toxicity and immunogenicity profiles of fluorescein conjugates may differ, it is reasonable to expect that the potential risk for the development of neutralizing antibodies within the given time window of days to few weeks for combined AML and HSPC targeting will be low [60], an assumption that will need future testing for each specific format.

While our study covers several benefits of using AdCAR T-cells and respective linkers, it also shows limitations. Clearly, high-risk AML patients with most complex genetic alterations in their leukemia cells are those with the highest unmet therapeutic need. Further preclinical work, e.g. with patient derived xenograft models, is necessary to determine if our approach will also be efficient in these challenging diseases settings.

Moreover, bringing multiple therapeutic elements at the same time in clinical development poses a difficulty. It may be necessary and more feasible to take a stepwise approach, initially developing AdCAR T-cells in combination with a single linker, and subsequently adding others, similar to ongoing trials with other AdCAR T-cells and bispecific antibodies [12, 65]. Also, while therapeutic efficacy can be evaluated in surrogate in vitro and in vivo assays, complex combinatorial toxicity evaluation presents a substantial challenge with species-specific antigen binders. Nevertheless, we believe the present study broadens the foundation for the clinical development of AdCAR T-cell therapies in complex diseases such as AML.

### Reporting summary

Further information on research design is available in the Nature Portfolio Reporting Summary linked to this article.

## Supplementary information

Supplementary Information

## Data Availability

The Supplementary Material contains detailed information on statistical analysis, isolation of human cells, flow cytometry and antibodies (Table S2), cytotoxicity assays, live-cell imaging, histology, and PK-PD in vivo studies of Db-FM. The data that support the findings of this study are available from the corresponding author upon reasonable request.
