# Supplementary material for: Efficient combinatorial adaptor-mediated targeting of acute myeloid leukemia with CAR T-cells
Source: Leukemia. 2024 Sep 18;38(12):2598–613. doi: 10.1038/s41375-024-02409-1 (PMC11588662; doi:10.1038/s41375-024-02409-1)
Supplement: Supplementary file 1 — Supplementary Information [file 41375_2024_2409_MOESM1_ESM.docx]

Supplementary Materials for

Efficient Combinatorial Adaptor-Mediated Targeting of

Acute Myeloid Leukemia with CAR T-Cells

**Authors:**

Laura Volta^1,2^, Renier Myburgh^2^, Christian Pellegrino^1,2^, Christian Koch^2,5^, Monique Maurer^2^, Francesco Manfredi^2^, Mara Hofstetter^2^, Anne Kaiser^2^, Florin Schneiter^5^, Jan Müller^2^, Marco M. Buehler^3^, Roberto De Luca^6^, Nicholas Favalli^6^, Chiara F. Magnani^2,4^, Timm Schroeder^5^, Dario Neri^1,6, #^, Markus G. Manz^2,4,*,#^

*Corresponding author. Email: markus.manz@usz.ch

**Supplementary Methods**

**T-cell isolation and CAR T-cell production**

Healthy donors’ buffy coats were acquired from the Zürich blood donation service (Blutspende Zürich, Zürich, Switzerland). PBMCs were enriched by density gradient centrifugation (Ficoll-Paque Plus, GE healthcare). T-cells were then negatively isolated with EasySep^TM^ beads (Human T cell isolation kit, STEMCELL Technologies) and cryopreserved until use. Purified T-cells were activated with CD3/CD28 Dynabeads (Thermo Fischer Scientific) in a 1:1 bead-to-cell ratio and cultured at a density of 1-2x10^6^ cells/ml in T-cell medium, comprised of advanced RPMI media supplemented with 10% FBS, penicillin/streptomycin (100 U/ml/100 µg/ml; Gibco, Thermo Fisher Scientific), 1x Glutamax^TM^ (Gibco) and 100 U/ml recombinant human IL-2 (Peprotech). On the following day, cells were transduced with concentrated lentiviral particles with the addition of hexadimethrine bromide (Sigma-Aldrich) at a final concentration of 8 µg/ml followed by centrifugation.

**Protein Characterization**

Quality control of the proteins was performed by SDS-PAGE, size exclusion chromatography, and mass spectrometry. LC-MS was performed on a Waters Xevo G2-XS Qtof instrument (ESI-ToF-MS) coupled to a Waters Acquity UPLC H-Class System using a 2.1x50 mm Acquity BEH300 C4 1.7mm column (Waters). Gradient method and solvents were prepared as previously described^1^.

**Affinity measurements**

Affinity measurements were performed by SPR using a BIAcore X100 instrument (Cytiva) on a CM5 chip coated with recombinant His-tagged extracellular domains of CD117 or CD33 (both Sino Biological). Site-specifically conjugated Dbs were injected as serial dilutions in a concentration range from 250 to 7.8 nM. The chip was regenerated using HCl 10 mM. MOLM14-CD117^high^ GFP^+^-Luc^+^ cells were centrifuged and washed in FACS buffer. Cells were stained with serial dilutions of Db-FM targeting CD33 and CD117 for 2h at 4°C to avoid internalization^2^ and stained with anti-FITC APC antibody before acquisition.

**Cell lines**

Kasumi-1 cells (CRL-2724, ATCC), MOLM13 (ACC 554, DSMZ) and MOLM14 (ACC 777, DSMZ) cells were cultured in RPMI-1640 supplemented with 20% FBS and 1% penicillin/streptomycin (all Gibco, Thermo Fisher Scientific). NB4 cells (ACC 207, DSMZ), NOMO-1 cells (ACC 542, DSMZ), and PLB985 cells (ACC 139, DSMZ) were cultured in RPMI supplemented with 10% FBS and 1% penicillin/streptomycin (R10). TF-1 cells (CRL-2003, ATTC) were cultured in RPMI supplemented with 10% FBS and 1% penicillin/streptomycin and 2 ng/ml recombinant human GM-CSF (Peprotech). UT-7 cells (ACC 137, DSMZ) were cultured in RPMI supplemented with 20% FBS and 1% penicillin/streptomycin and 5 ng/ml recombinant human GM-CSF. THP-1 cells (ACC 16, DSMZ) were cultured in RPMI supplemented with 10% FBS, 1% penicillin/streptomycin and 50nM 2-mercaptoethanol. OCI-AML3 cells (ACC 582, DSMZ), HL-60 cells (CCL-240, ATCC) and KG-1 cells (CCL-256, ATTC) were maintained in Iscove's Modified Dulbecco's Medium (IMDM; Gibco), supplemented with 20% FBS and 1% penicillin/streptomycin. K562 cells (ACC 10, DSMZ) and MV-4-11 cells (CRL-9591, ATTC) were cultured in IMDM supplemented with 10% FBS and 1% penicillin/streptomycin.

**Transduction of cell lines**

For lentiviral transduction of CD117 into cell lines, a truncated version of the GNNK^+^ isoform of the CD117 gene, without intracellular signaling domain was cloned into a lentiviral vector (pCLX-UBI, kindly provided by Dr. Patrick Salmon, University of Geneva) by gateway cloning. Lentiviral particles were produced using HEK293T cells, and cell transduction was performed following previously established protocols^3,4^. MOLM14 WT and CD33KO cells were kindly provided by Dr. Saar Gill (University of Pennsylvania)^5^. Cells were transduced with concentrated lentiviral particles carrying the CD117 gene and expanded. MOLM14 WT and CD33KO were bulk-sorted for CD117 expression with BD FACSAria cell sorter, and single clones were selected by limiting dilution methods. The resulting populations were expanded, evaluated for unaltered growth kinetics, and cryo-preserved.

**Flow cytometry-based assays**

Dead cells were excluded from the analysis by staining with DAPI (0.25 mg/ml final concentration, Thermo Fisher Scientific) or Zombie Aqua (Biolegend). Cells were subsequently stained with fluorescently labeled antibodies in FACS buffer (PBS, 2% FBS, 2 mM EDTA). Antibodies used in this study are indicated in **Table S2**. Samples were acquired on a BD LSR Fortessa II flow cytometer (Becton Dickinson). FlowJo software (v10.0.7, Treestar) was used for data analysis and presentation.

**In vitro cytotoxicity assays and cytokine quantification**

Cell lines were maintained in the appropriate culture medium until set-up of the killing assay. Mononuclear (MNC) or CD3^+^/CD19^+^double depleted BM or PB cells isolated from AML patients at initial diagnosis were thawed on the same day of the assay setup. AdFITC-CAR T-cells and target cells were co-cultured in T-cell medium in 96-well round bottom plates for up to 72h at indicated effector-to-target ratios. Dilution series of antibody adaptors were added to the cell solution and incubated until readout. Prior to antibody staining, cell-free supernatant was harvested and stored at -20°C until analysis. Cell lysis was assessed by flow cytometry using BD LSR Fortessa II cell analyzer (Becton Dickinson) at the indicated time points. Percentage of specific lysis was calculated as follows:


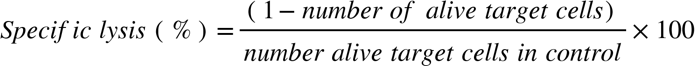


For proliferation assays, effector T- and AdFITC-CAR T-cells were labeled with 2μM CellVue Claret Red Dye fluorescent membrane dye (Sigma-Aldrich) according to manufacturer’s instructions and resuspended in T-cell medium with and without addition MOLM14-CD117^high^ GFP^+^Luc^+^ and/or 10nM CD117 Db-FM. Proliferation was assessed by CellVue Claret dilution by flow cytometry after 24, 48 and 72h. Unless otherwise noted, all cytotoxicity experiments were performed using three different healthy-donor-derived AdFITC-CAR T-cells or T-cells, each plated in duplicate wells.

The levels of secreted IFN-γ and IL-2 in the supernatant of *in vitro* cocultures were quantified by Enzyme-linked Immunosorbent Assay (ELISA) according to the manufacturer’s instructions (ELISA MAX Deluxe Set, Biolegend). Absorbance at 450nm was read on a Mini ELISA Plate Reader™ (Biolegend).

**Live-cell imaging**

Time-lapse experiments were performed at 37°C, 5% CO_2_ in R10 growth medium supplemented with propidium iodide (2µM, eBioscience) on µ-Slides with four wells (Ibidi®). Self-adherent silicone polymer microgrid arrays (50x50µm, Microsurfaces) were glued to the bottom of each well to allow for separate observation of individual lytic events. Effector cells were stained with CellVue Claret Far Red dye according to the manufacturer’s instructions prior to co-incubation. MOLM14-CD117^high^, MOLM14-CD117^mid^ or MOLM14 cells were used as target cells at an E:T ratio of 1:1 with 4×10^4^ cells per well. CD117- and CD33-DbFM were added at 9nM final concentration. Time-lapses were conducted on a Nikon-Ti2-E Eclipse equipped with linear-encoded motorized stage, Orca Flash 4.0 V2 (Hamamatsu), a Spectra X fluorescent light source (Lumencor), a 10× CFI Plan Apochromat λ objective (NA 0.45) and an incubation chamber. Appropriate filter sets (all AHF) were chosen to detect fluorescence: CellVue Claret Far Red (620/60; 660LP; 700/75) and propidium iodide (550/32; 585LP; 605/15). Bright field images were acquired using white light emitted by the Spectra X and a custom-made motorized mirror controlled by Arduino UNO Rev3 (Arduino). Typically, 16 positions per microgrid were acquired at 10min time intervals for bright field, and 10 or 30min intervals for both PI and CellVue, for up to 72h. Single-cell tracking was performed using custom-written software^6–8^. For the in vitro experiments shown in **Fig. S5A**, bright-field images were taken with an upright epifluorescence microscope (DM5500-B, Leica) at 5× magnification (HC PL FLUOTAR 5x/0.15 Leica objective). Leica LAS-X software was used for microscope control, image acquisition and analyses.

**Pharmacokinetics of Db-FM *in vivo***

NSG mice received 25 μg CD117 Db-FM i.v. or i.p. (two mice per route of injection and time point) in 65 μl total volume. Blood was sampled from all the mice 1min after injection and taken terminally at 5, 10, 30, 60, and 120 min post-injection. Blood samples were incubated on ice for 2h, then centrifuged for 20min at 12000 xg and serum was stored at −20°C until analysis. CD117 Db-FM concentration in PB samples was determined with an in-house ELISA. Briefly, 96-well plates (Nunc MaxiSorp, Biolegend) were coated overnight with recombinant His-tagged CD117 extracellular domain (10nM in 100 μl PBS, SinoBiological). The next day, plates were incubated with serum samples in diluent A (1:4 ratio, Biolegend). Bound CD117 Db-FM was detected with Protein A-biotin (2μg/ml in PBS, Sigma Aldrich) and avidin-horseradish peroxidase (Biolegend) for amplification of the signal. Concentration of serum samples was interpolated from standard curves obtained by diluting CD117 Db-FM in healthy NSG mouse serum and diluent A.

**Db-FM-mediated AdFITC-CAR T-cell activation in the absence of target cells *in vivo***

Mice were administered 10^7^ AdFITC-CAR T-cells, with or without subsequent administration of 25 μg anti-CD117 Db-FM adaptors (twice daily, i.p.). After 48h, at terminal analysis, spleen, blood, and bone marrow were collected for flow analysis. Bone marrow cells derived from both femora and tibiae femur were resuspended in 1ml single-cell suspension in FACS buffer and 200 μl were analyzed. Single-cell suspensions of spleen (1 ml) and 150 ml blood were obtained following incubation in red blood cell lysis buffer (Biolegend). Additionally, blood samples were incubated on ice for 2h, then centrifuged for 20 min at 12000 xg and serum was stored at −20°C until cytokine analysis. IFN-γ and IL-2 levels in mouse sera were measured using ELISA following the manufacturer's protocol (ELISA MAX Deluxe Set, Biolegend).

**Determination of Db-FM on-tumor residence time**

Sublethally irradiated NSG mice (100 cGy; RS-2000 irradiator, Rad Source) were engrafted with 0.1×10^6^ MOLM14-CD117^high^ GFP^+^Luc^+^ cells i.v. injected. After 10 days, presence of tumor was assessed by bioluminescence imaging (BLI). Mice were anesthetized before imaging and administered i.p. 10 μl/g of body weight IVISbrite™ D-Luciferin (diluted at 15 g/l in D-PBS, PerkinElmer). Images were acquired on IVIS® Lumina X5 in vivo imaging system (PerkinElmer) 5 min after injection using 40sec exposure, binning 4. BLI data were analyzed with Living Image software (PerkinElmer), and total flux was recorded from ROIs encompassing the mouse body. On day 10, mice were administered with 50 μg CD33 and/or CD117 Db-FM adaptors i.v. or i.p. at the indicated doses. At terminal analysis, bone marrow collected from femora and tibiae was pooled and resuspended in 1ml single cell suspension in FACS buffer prior to staining. Presence of Db-FM on tumor (hCD45^+^GFP^+^) cell surface was measured by flow cytometry staining with anti-FITC APC antibodies.

**Histology and Immunohistochemistry**

For histological analysis, mouse femora were fixed in 4% buffered formalin, followed by EDTA decalcification with MoL-DECALCIFIER on a DecalMATE instrument (Milestone Medical), tissue processing on a Leica PELORIS 3 tissue processor (Leica Biosystems) and embedded in paraffin. Tissue sections (2-3 μm) were mounted on SuperFrost Plus slides (ThermoFisher Scientific) and stained with hematoxylin and eosin (H&E). Immunohistochemistry (IHC) was performed using a monoclonal anti-CD117 antibody (clone YR145, rabbit monoclonal, Cell Marque), 1:200 dilution, 32 min incubation, OptiView DAB detection kit (Ventana Medical Systems) on a Ventana Benchmark ULTRA system (Ventana Medical Systems). Specimens were analyzed by light microscopy and digitalized with a Nano Zoomer C9600 scanner (Hamamatsu Photonics).

**Mathematical Modeling**

MOLM14 doubling time *in vivo* was determined by fitting tumor growth with an exponential model^9^, where cells grow at a constant rate (*r*). To quantify tumor growth rate, the doubling time *DT* is then defined as:


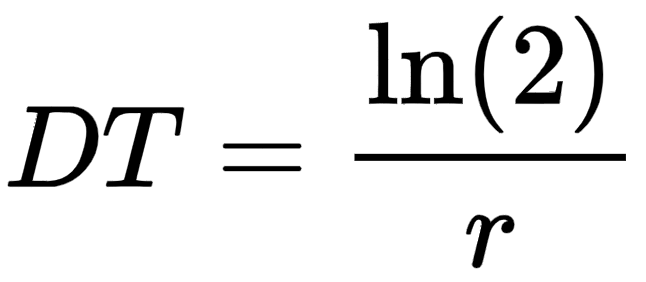


Pharmacokinetic curves were interpolated using an exponential decay function after i.v. bolus injection or Bateman’s function when Db-FM were administered i.p. Curve fitting of percentage cell lysis was performed with a bell-shaped dose-response curve. On-tumor CD33 and CD117 Db-FM concentrations were interpolated with five-parameter logistic equations (5PL). GraphPad Prism 10 software was used for data analysis.

**Statistical Analysis**

Data are reported as mean ± standard deviation (SD) unless otherwise noted in figure legends. Statistical analyses were conducted with GraphPad Prism (version 10). Variances observed were similar across experimental groups in the results reported. *P* value of <0.05 was considered significant, and the significance is denoted by **p* < 0.05, ***p* < 0.01, ****p* < 0.001, *****p* < 0.0001.

**Supplementary References**

1 Pellegrino C, Favalli N, Sandholzer M, Volta L, Bassi G, Millul J *et al.* Impact of Ligand Size and Conjugation Chemistry on the Performance of Universal Chimeric Antigen Receptor T‑Cells for Tumor Killing. *Bioconjugate Chem* 2020; **31**: 1775–1783.

2 Nixdorf D, Sponheimer M, Berghammer D, Engert F, Bader U, Philipp N *et al.* Adapter CAR T cells to counteract T-cell exhaustion and enable flexible targeting in AML. *Leukemia* 2023; : 1–13.

3 Myburgh R, Kiefer JD, Russkamp NF, Magnani CF, Nuñez N, Simonis A *et al.* Anti-human CD117 CAR T-cells efficiently eliminate healthy and malignant CD117-expressing hematopoietic cells. *Leukemia* 2020; **34**: 2688–2703.

4 Magnani CF, Myburgh R, Brunn S, Chambovey M, Ponzo M, Volta L *et al.* Anti-CD117 CAR T cells incorporating a safety switch eradicate human acute myeloid leukemia and hematopoietic stem cells. *Mol Ther - Oncolytics* 2023; **30**: 56–71.

5 Kim MY, Yu K-R, Kenderian SS, Ruella M, Chen S, Shin T-H *et al.* Genetic Inactivation of CD33 in Hematopoietic Stem Cells to Enable CAR T Cell Immunotherapy for Acute Myeloid Leukemia. *Cell* 2018; **173**: 1439-1453.e19.

6 Hilsenbeck O, Schwarzfischer M, Skylaki S, Schauberger B, Hoppe PS, Loeffler D *et al.* Software tools for single-cell tracking and quantification of cellular and molecular properties. *Nat Biotechnol* 2016; **34**: 703–706.

7 Hoppe PS, Schwarzfischer M, Loeffler D, Kokkaliaris KD, Hilsenbeck O, Moritz N *et al.* Early myeloid lineage choice is not initiated by random PU.1 to GATA1 protein ratios. *Nature* 2016; **535**: 299–302.

8 Loeffler D, Wehling A, Schneiter F, Zhang Y, Müller-Bötticher N, Hoppe PS *et al.* Asymmetric lysosome inheritance predicts activation of haematopoietic stem cells. *Nature* 2019; **573**: 426–429.

9 Shackney SE. A computer model for tumor growth and chemotherapy, and its application to L1210 leukemia treated with cytosine arabinoside (NSC-63878). *Cancer Chemother Rep* 1970; **54**: 399–429.

**Supplementary Figure 1**

**
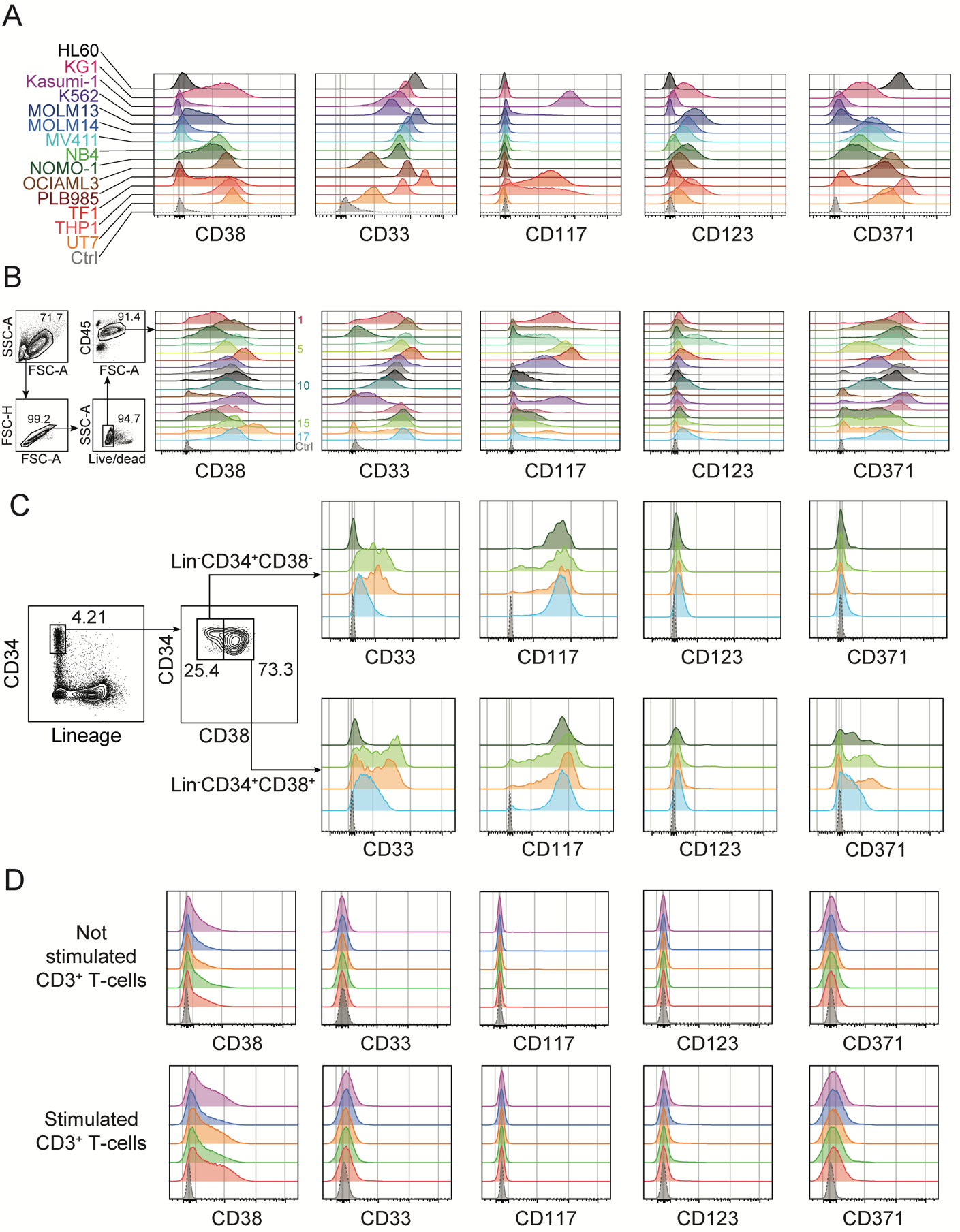
**

**Supplementary Figure 1.** **Cell surface expression of immune-therapeutically targetable antigens on human AML cell lines, patient-derived AML blasts, healthy donor bone marrow cells, and healthy donor T-cells.**

**(A)** Flow cytometry histograms showing cell surface expression of human CD33, CD38, CD117, CD123, and CD371 on 14 human AML cell lines. Expression was compared to an unstained control (HL60, dashed grey line). **(B)** Representative FC plots showing the gating strategy applied to 17 AML/MDS/CMML patient CD3/CD19 depleted peripheral blood and bone marrow samples. Cell surface expression of human CD33, CD38, CD117, CD123, and CD371 on gated CD45^dim^ blast cells are reported as flow cytometry histograms. Expression of the antigens is compared to an unstained patient sample (dashed grey line) used as threshold level. **(C)** Representative flow cytometry plots displaying the gating strategy for analyzing healthy donor bone marrow samples (left panel). Cell surface expression of human CD33, CD123, CD117, and CD371 on Lin^-^ (CD3, CD14, CD16, CD19, CD20, CD56) CD34^+^CD38^-^ and Lin^-^CD34^+^CD38^+^ cells from 3 bone marrow samples and one CD34-enriched bone marrow sample (in blue) is shown in flow cytometry histograms (right panel). Expression was compared to an unstained BM sample (dashed grey line). **(D)** FC histograms showing cell surface expression of human CD33, CD38, CD117, CD123, and CD371 on 5 healthy donor CD3^+^ T-cells, either not-stimulated (top panel) or stimulated for 24h in IL-2 (bottom panel). Expression was compared to an unstained unstimulated T cell sample (dashed grey line).

**Supplementary Figure 2**

**
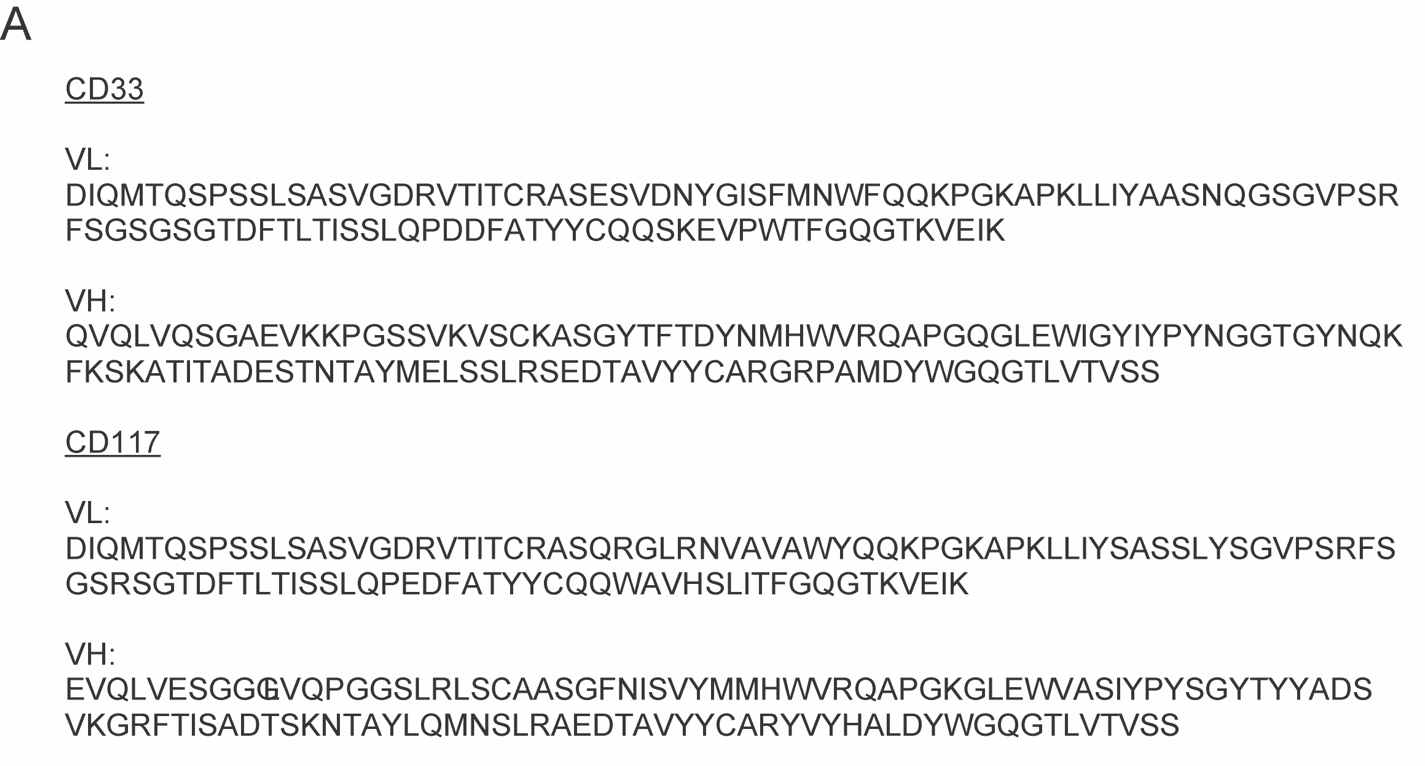
**

**Supplementary Figure 2.** **Sequences of antibodies against CD33 and CD117.**

**(A)** Variable light (VL) and variable heavy (VH) sequences of the parental anti-CD33 and CD117 antibodies.

**Supplementary Figure 3**

**
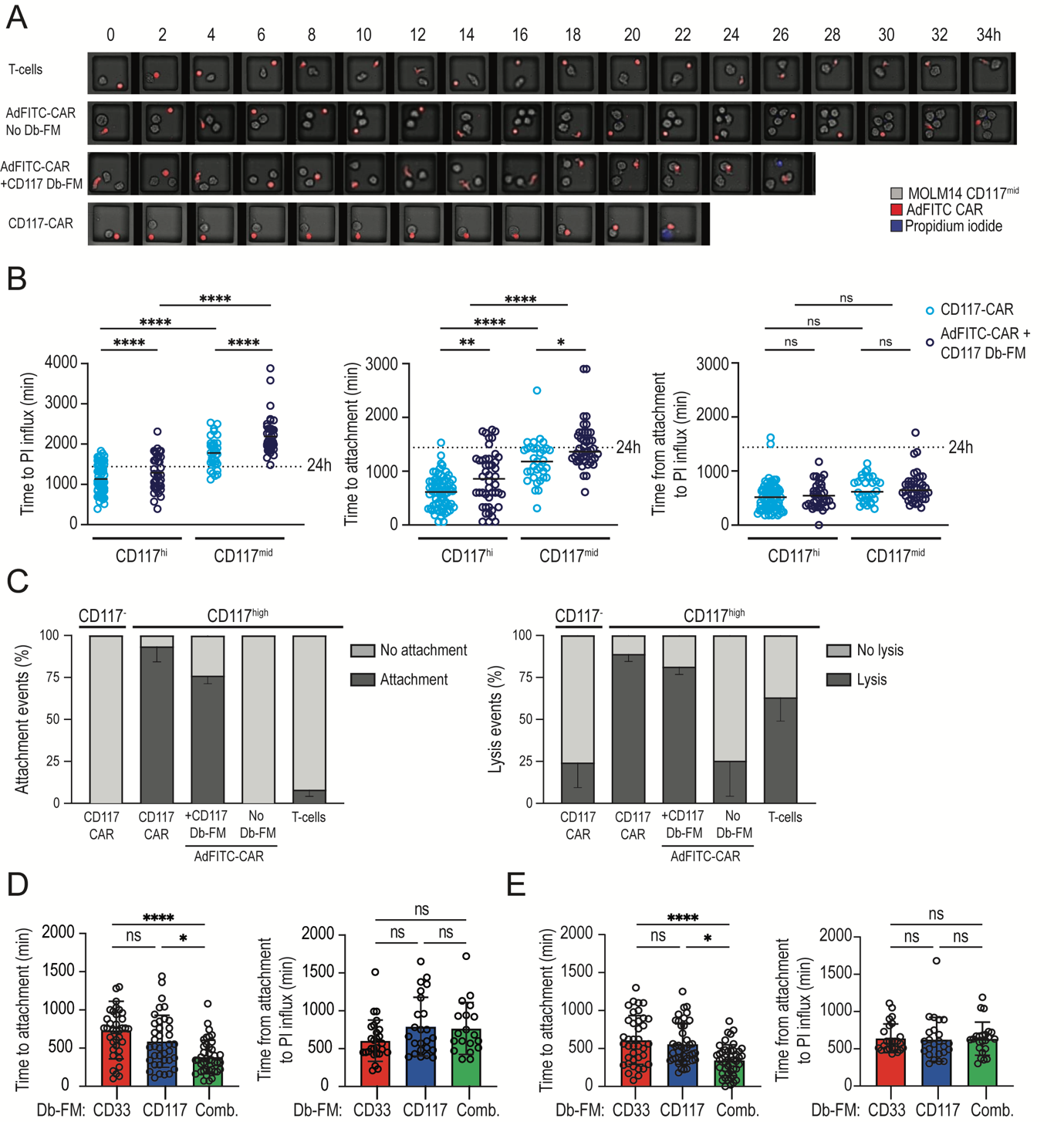
**

**Supplementary Figure 3**. **Live cell imaging of tumor cells with increasing target-antigen density and various effector cells.**

**(A)** Representative time-lapse images of MOLM14-CD117^high^ cells (grey) and untransduced T-cells, AdFITC-CAR T-cells (with or without 9nM anti-CD117 Db-FM), or direct anti-CD117 CAR T-cells (effector cells in red). Cells were cocultured at an E:T=1:1 for up to 72h on microgrids to allow for quantification of single attachment and lysis events. Target cell lysis is marked by PI influx (blue). **(B)** AdFITC-CAR T-cells and anti-CD117 Db-FM were compared to direct anti-CD117 CAR T-cells with respect to their lysis abilities against MOLM14-CD117^mid^ and MOLM14-CD117^high^ cells. The time to PI influx (left panel) was further divided into two components: the time to target-cell attachment (middle) and the time from effector-cell attachment to target-cell lysis (right). **(C)** Quantification of target-cell interaction showed effective and antigen-specific target cell attachment within 24h (left panel). After attachment, no lysis of target cells was observed in the absence of target antigen or adaptor molecules (right panel). T-cells without CAR showed some activation via alloreactivity over 72h. **(D, E)** Time-lapse imaging showing attachment and time to lysis of MOLM14-CD117^mid^CD33^+^ cells in two independent experiments, each with AdFITC-CAR T-cells derived from two distinct healthy donors, in combination with CD33 and/or CD117 Db-FM. Sustained effector on the target-cell attachment but not the time from target-attachment to lysis was significantly shorter with the combinatorial adaptor approach when compared to single targeting. Statistical analysis was performed with one way ANOVA; * p <0.05; ** p <0.01; *** p <0.001; **** p< 0.0001.

**Supplementary Figure 4**


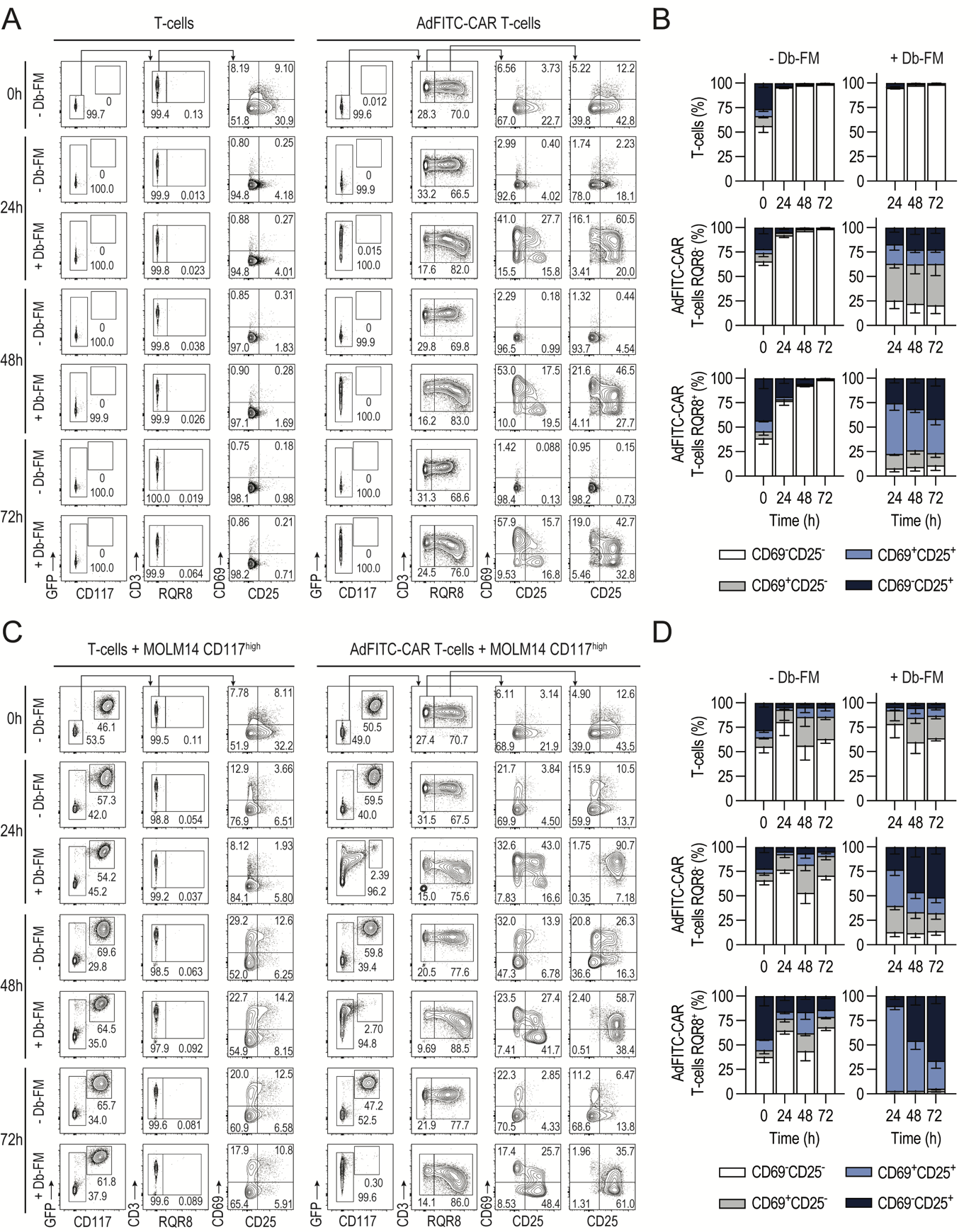


**Supplementary Figure 4. Activation status of AdFITC-CAR T-cells in absence and presence of CD117 Db-FM with or without target cells.**

**(A)** Representative flow cytometry plots showing the activation status of RQR8^+^ and RQR8^-^ subpopulations of T-cells and AdFITC-CAR T-cells cultured with or without 10nM anti-CD117 Db-FM in absence of target cells at 0, 24, 48 and 72h. **(B)** Quantification of the CD69 and CD25 single- and double-positive fractions. **(C, D)** T-cells or AdFITC-CAR T-cells were co-cultured with MOLM14-CD117^high^ cells at an E:T=1:1 in presence or absence of anti-CD117 Db-FM and activation status was evaluated at 0, 24, 48 and 72h (representative FC in **C**, quantified in **D**). **(A-D)** Experiment performed in duplicates with AdFITC-CAR T-cells and expanded T-cells derived from 3 matched healthy donors (mean ± SD).

**Supplementary Figure 5**

**
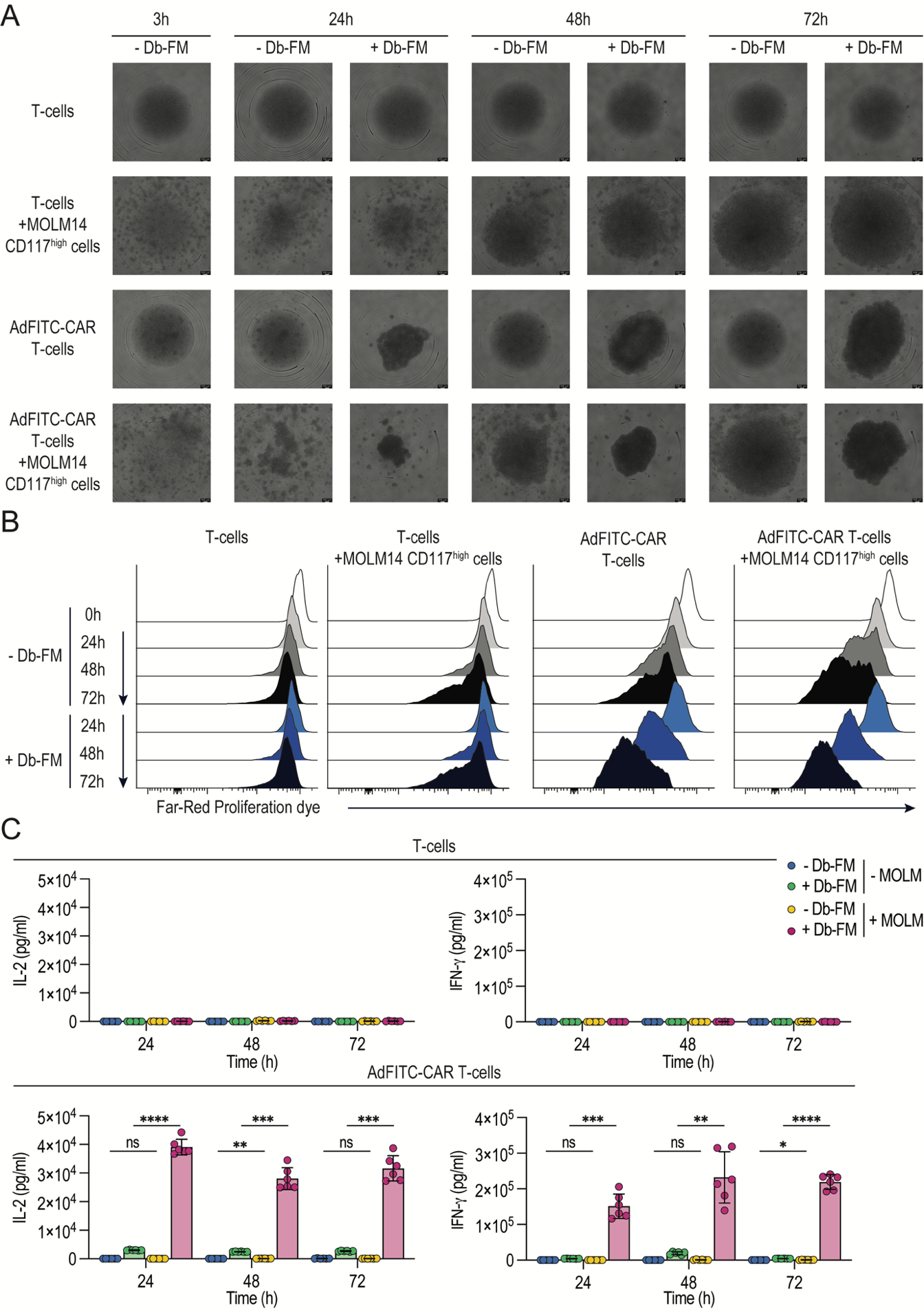
**

**Supplementary Figure 5. Clustering, proliferation and cytokine release of AdFITC-CAR T-cells in absence or presence of CD117 Db-FM with or without target cells.**

T-cells and AdFITC-CAR T-cells were cultured for up to 72h with or without MOLM14-CD117^high^ cells (E:T=1:1) in presence or absence of 10 nM anti-CD117 Db-FM. **(A)** Bright field images of representative wells were taken 3, 24, 48, and 72 hours after the beginning of the experiment. **(B)** Proliferation of T-cells or AdFITC-CAR T-cells in the indicated conditions by means of CellVue Far-Red dye dilution. (**C**) IL-2 and IFN-γ levels in supernatants after 24, 48, and 72h. Statistical analysis was conducted using two-way ANOVA; * p <0.05; ** p <0.01; *** p <0.001; **** p< 0.0001. **(A-C)** Experiment performed in duplicates with donor-matched AdFITC-CAR T-cells and expanded T-cells derived from 3 different healthy donors (mean ± SD).

**Supplementary Figure 6**

**
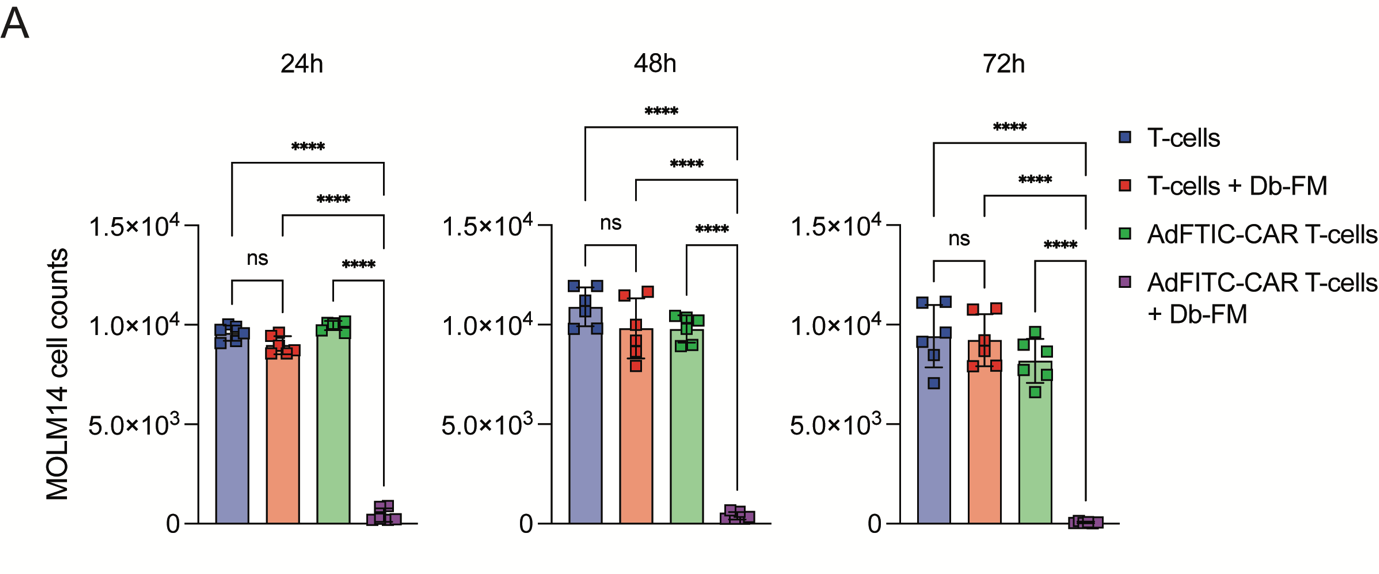
**

**Supplementary Figure 6. MOLM14-CD117^high^ cell lysis from AdFITC-CAR T-cells and T-cells in absence or presence of CD117 Db-FM.**

T-cells and AdFITC-CAR T-cells were incubated for up to 72h with MOLM14-CD117^high^ cells (E:T=1:1) in presence or absence of 10 nM anti-CD117 Db-FM. **(A)** Residual MOLM14 cells at the indicated time points after co-culture. Statistical analysis was conducted using one-way ANOVA; **** p< 0.0001. Experiment performed in duplicates with donor-matched AdFITC-CAR T-cells and expanded T-cells derived from 3 different healthy donors (mean ± SD).

**Supplementary Figure 7**


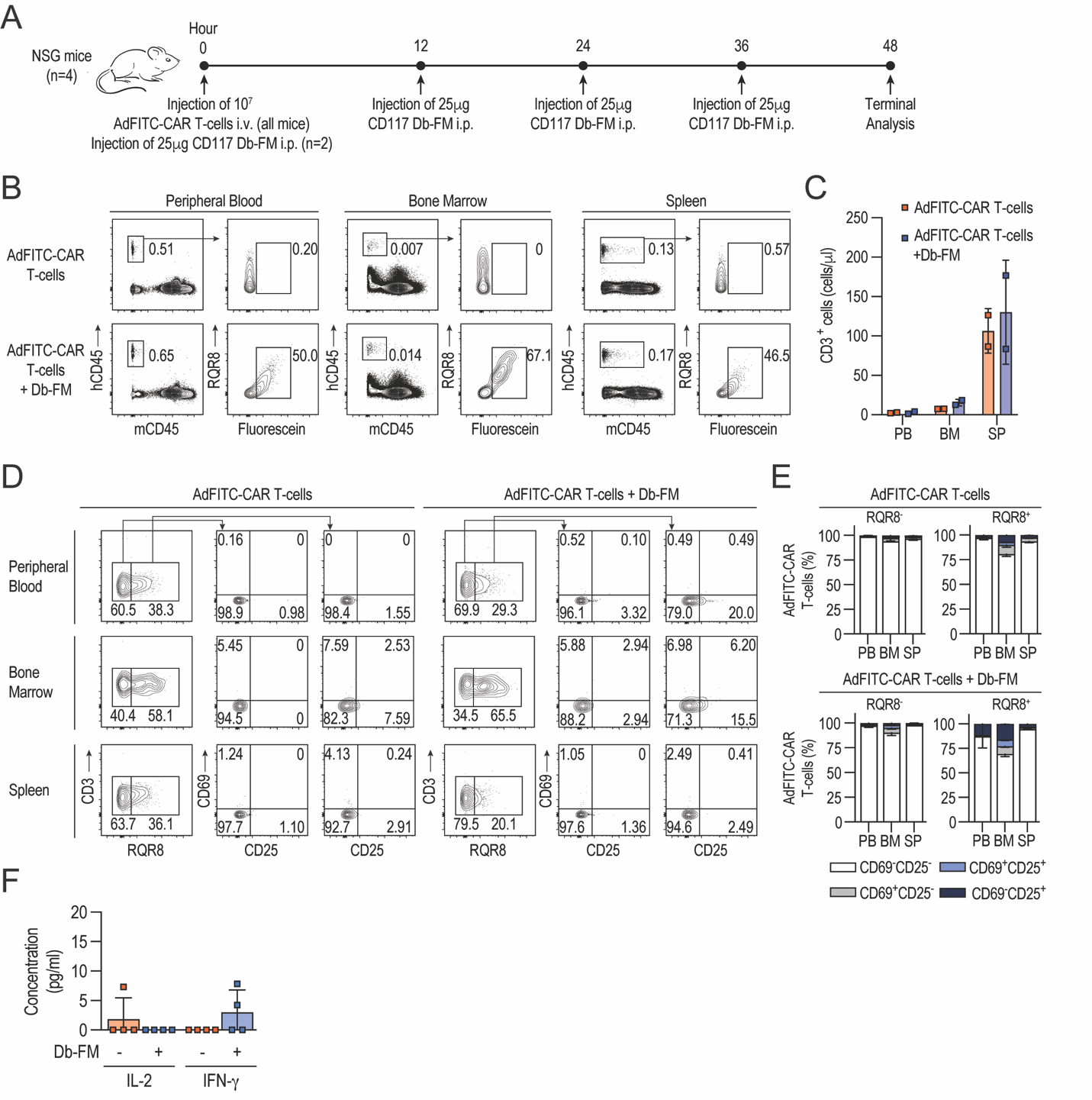


**Supplementary Figure 7. Activation status of AdFITC-CAR T-cells with or without Db-FM in absence of target cells *in vivo.***

**(A)** Schematic set-up to assess activation of AdFITC-CAR T-cells in presence of Db-FM but without target cells. NSG mice were injected with 10^7^ AdFITC-CAR T-cells i.v. and 25 μg CD117 Db-FM i.p. every 12h or no adaptors (n=2 mice per condition). **(B)** Representative flow cytometry plots showing the RQR8^+^fluorescein^+^ fraction of CAR T-cells (hCD45^+^) in peripheral blood, bone marrow and spleen at terminal analysis. **(C)** Absolute counts of hCD45^+^hCD3^+^ T-cells (mean ± SD) in the PB, BM and spleen (SP) at terminal analysis. **(D, E)** Activation status of AdFITC-CAR T-cells in different organs 48h post CAR T-cells injection (representative flow cytometry in **C**, quantified in **D**). **(F)** IL-2 and IFN-γ concentration in serum was determined by ELISA (measurements performed in duplicates).

**Supplementary Figure 8**


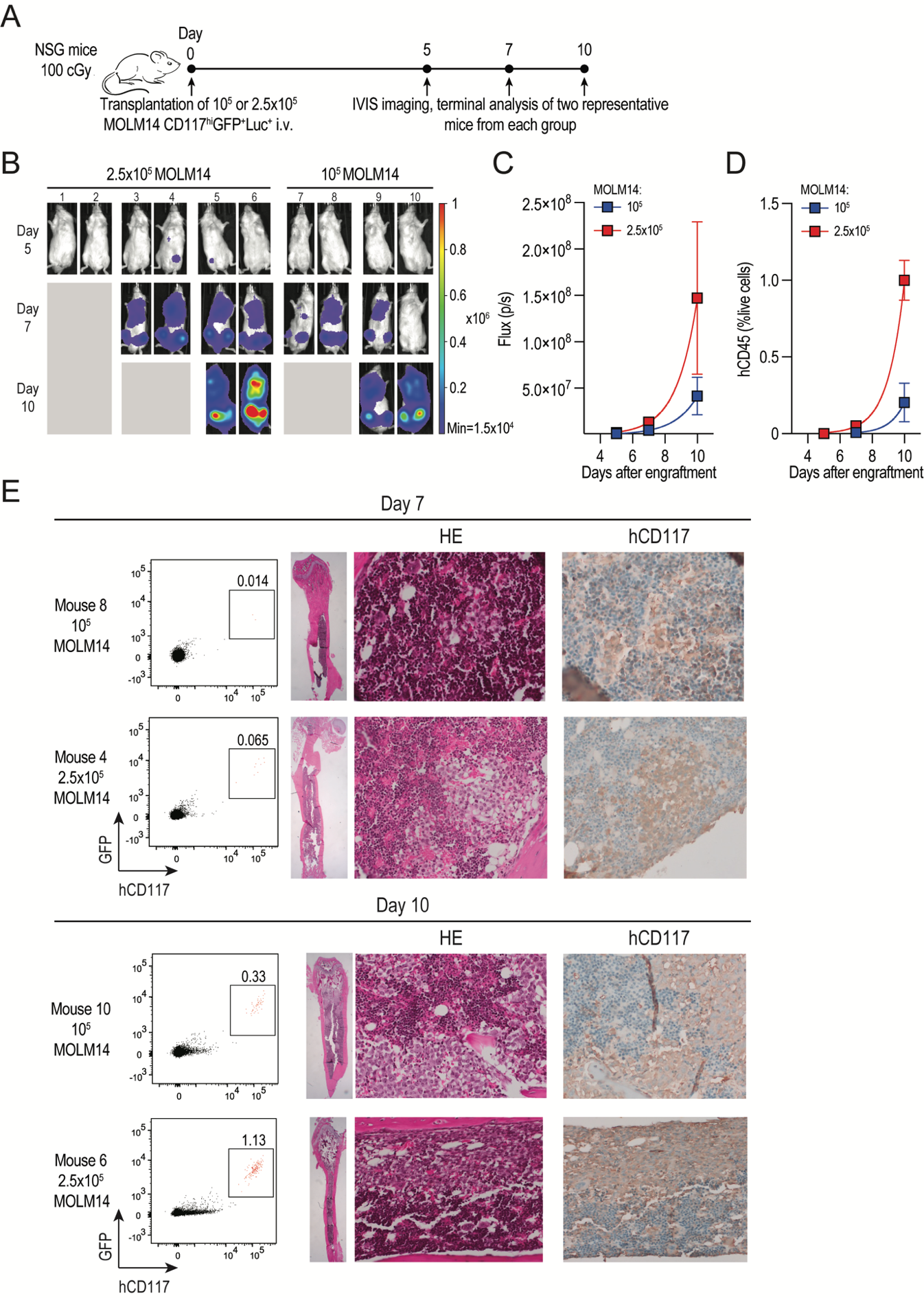


**Supplementary Figure 8. MOLM14-CD117^high^ growth kinetics *in vivo*.**

**(A)** Schematic outline of the experimental setup. Sub-lethally irradiated NSG mice (100cGy) were engrafted i.v. with 10^5^ or 2.5x10^5^ MOLM14-CD117^high^GFP^+^Luc^+^ cells. At days 5, 7, and 10 after tumor inoculation, engraftment was monitored by bioluminescence imaging, and the bone marrow of two representative mice per group was collected and analyzed by flow cytometry and immunohistochemistry. **(B)** Bioluminescence analysis of MOLM14 cell engraftment at the indicated time points. **(C)** Quantification of the bioluminescence flux of whole-body imaging of mice in **B** displayed exponential growth over time, exhibiting a modeled doubling time of 0.8 days for 2.5x10^5^ and 0.9 days for 1x10^5^ transplanted cells, respectively. **(D)** Percentages of hCD45^+^ cells within the live BM population increased exponentially over time with a modeled doubling time of 0.6 days following transplantation of 2.5x10^5^ or 1x10^5^ cells. **(E)** Flow cytometry plots, HE, and CD117 IHC staining of contralateral femora are shown for a representative mouse per group at the indicated days 7 and 10 after MOLM14 transplantation.

**Supplementary Figure 9**

**
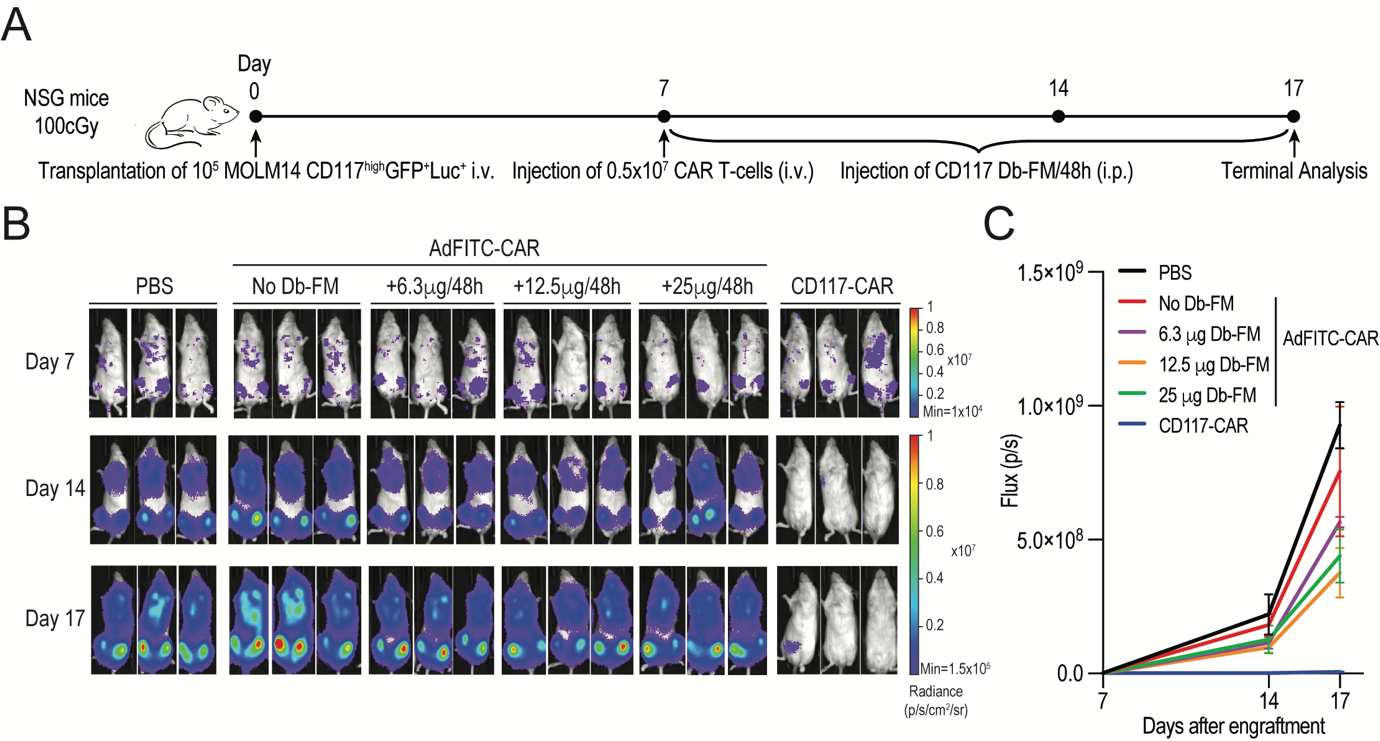
**

**Supplementary Figure 9. Effects of diabody administration frequency on therapeutic outcomes *in vivo*.**

**(A)** Experimental setup. Sub-lethally irradiated NSG mice were injected i.v. with 10^5^ MOLM14-CD117^high^GFP^+^Luc^+^ cells and after 7 days, successful engraftment was confirmed by BLI. Mice received injections of either 0.5x10^7^ direct CD117-CAR or AdFITC-CAR T-cells, along with decreasing doses of anti-CD117 Db-FM (25, 12.5, and 6.3 μg) administered i.p. every 48 hours (n=3 mice per group). **(B)** Bioluminescence analysis of MOLM14 cell engraftment at days 7, 14, and 17 in mice. The various conditions are indicated. **(C)** Evaluation of the bioluminescent flux in mice at the analyzed time points.

**Supplementary Figure 10**

**
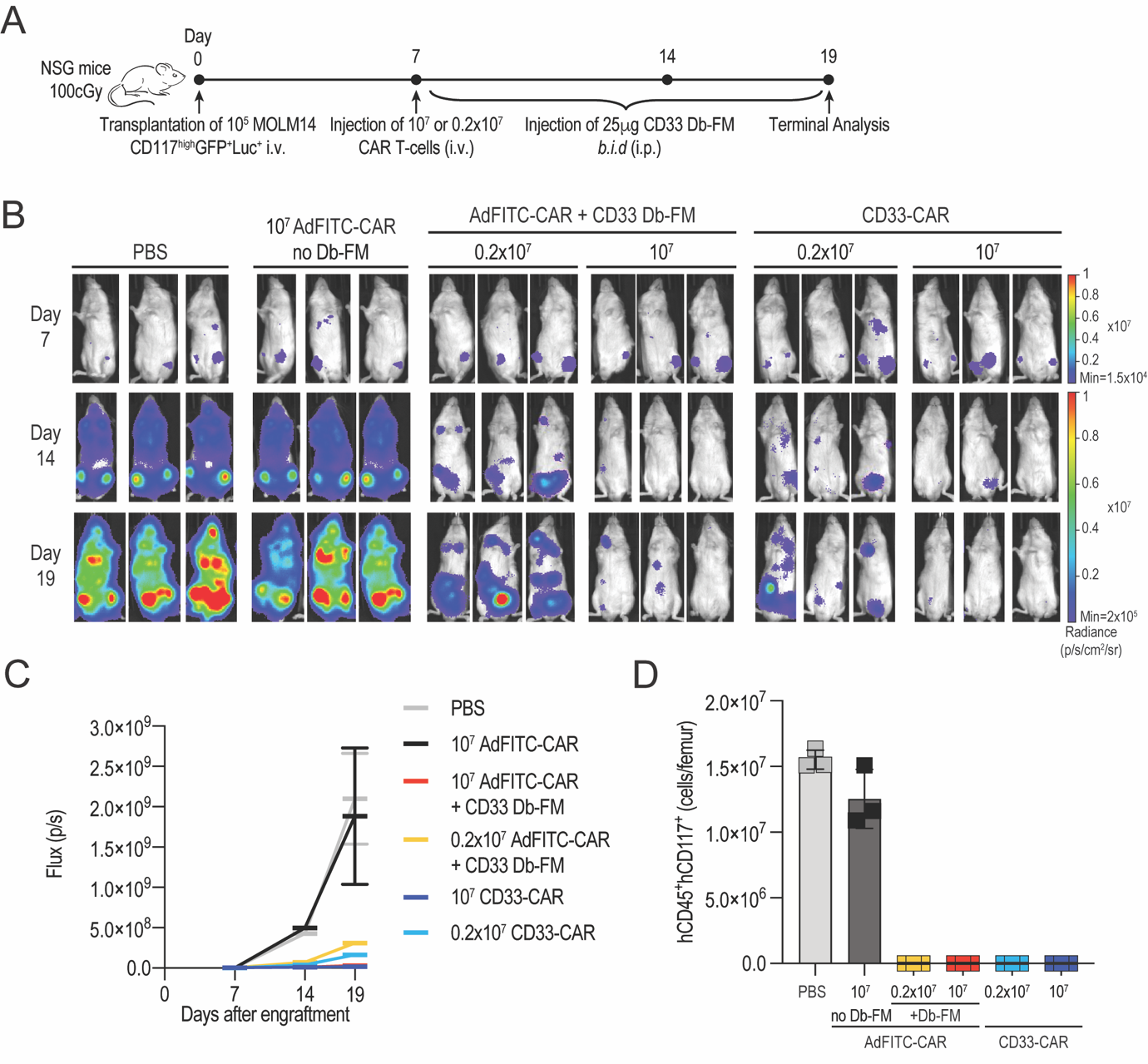
**

**Supplementary Figure 10. Effects of effector cell numbers on therapeutic outcome *in vivo*.**

**(A)** Schematic outline of the experimental setup (n=3 mice per group). Sub-lethally irradiated mice were engrafted with 10^5^ MOLM14-CD117^high^GFP^+^Luc^+^ cells. Seven days later, engraftment was confirmed by BLI, and mice were injected with 0.2x10^7^ or 10^7^ direct CD33-CAR or AdFITC-CAR T-cells i.v. and 25 μg CD33 Db-FM (i.p. every 12h) or no adaptors (negative controls). **(B)** Bioluminescence analysis of MOLM14 cell engraftment at days 7, 14, and 19 in mice under the indicated conditions. **(C)** Quantification of the bioluminescence flux of whole-body imaging of mice shown in **B**. **(D)** Absolute counts of hCD45^+^hCD117^+^ cells (mean ± SD) in the BM of single femora at terminal analysis.

**Supplementary Figure 11**


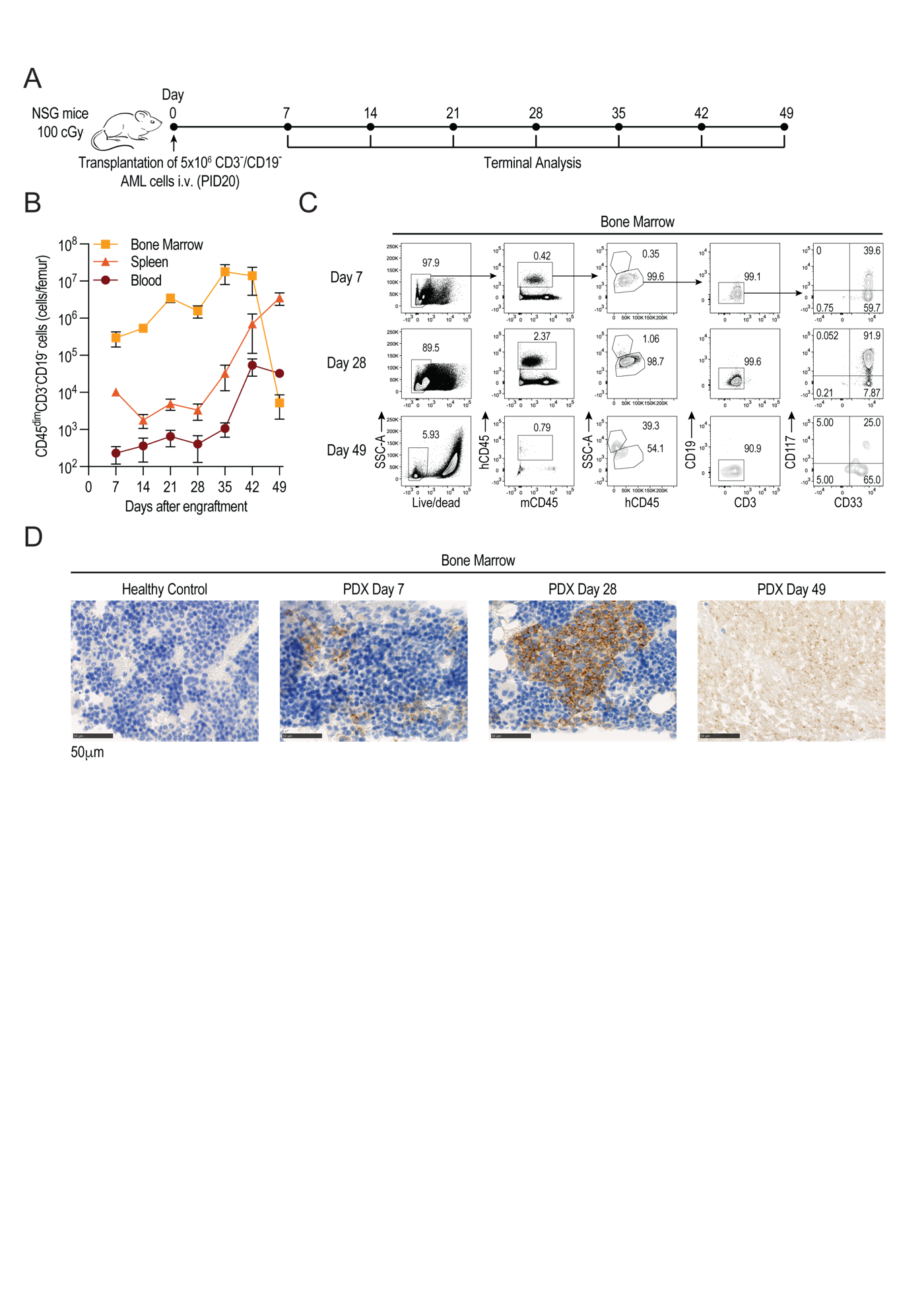


**Supplementary Figure 11. Growth kinetics of AML patient PID20 cells *in vivo*.**

**(A)** Schematic outline of the experimental setup. Sub-lethally irradiated NSG mice (100cGy) were engrafted i.v. with 5x10^6^ CD3/CD19 double-depleted PB cells isolated from AML patient PID20. After tumor inoculation, engraftment from three representative mice was analyzed weekly by flow cytometry and IHC in bone marrow, blood, and spleen. **(B)** Quantification of the absolute counts of AML patient cells (hCD45^dim^CD3^-^CD19^-^) isolated from BM of a femur, 150 μl of blood, and spleen at terminal analysis. Counts refer to a final resuspension volume of 1ml. **(C)** Representative flow cytometry plots of BM are shown for a representative mouse per group at the indicated days after transplantation. **(D)** Representative hCD45 IHC staining of the contralateral femora of the mice shown in **C**.

**Supplementary Figure 12**


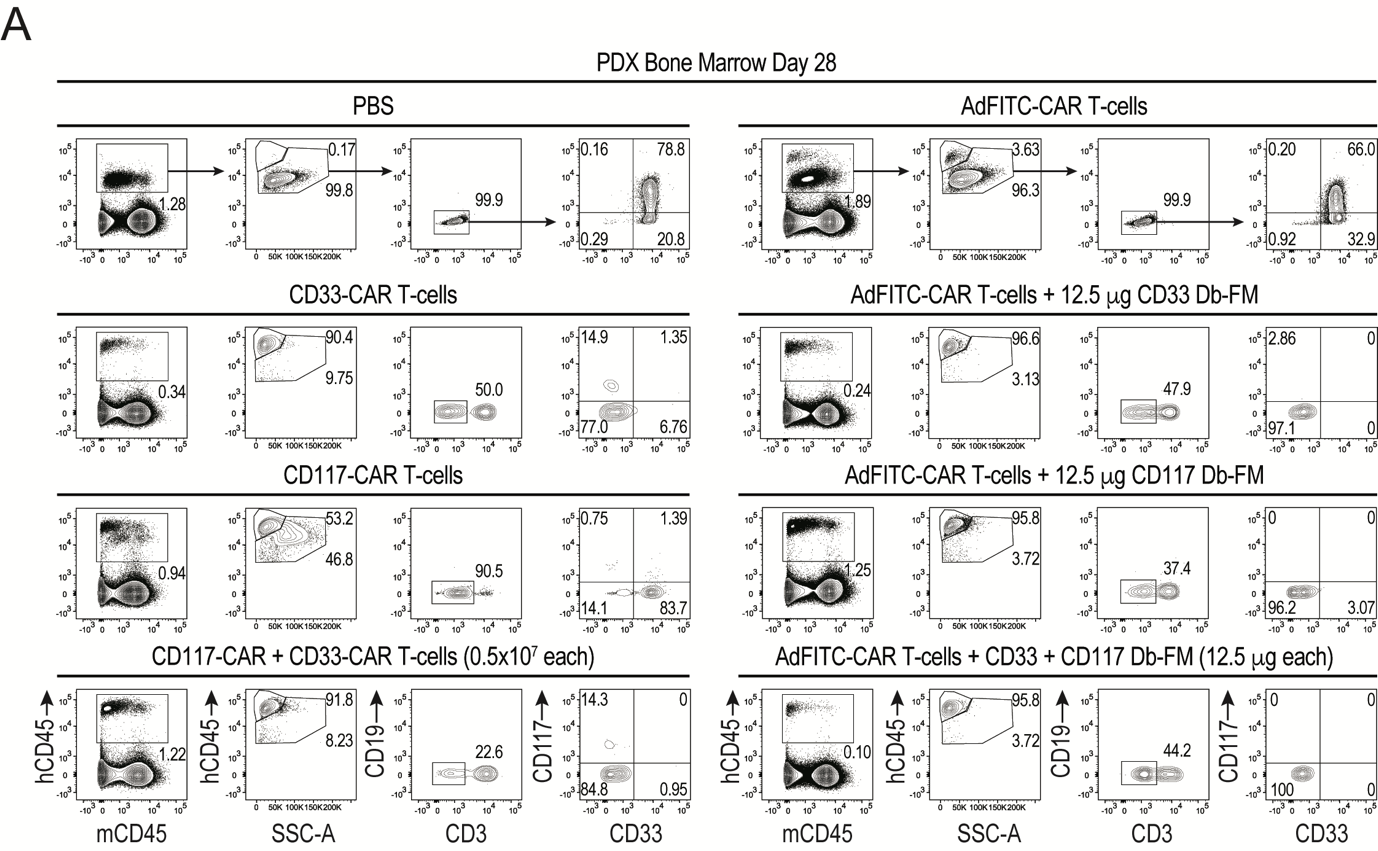


**Supplementary Figure 12. Gating strategy of mice engrafted with primary patient AML cells and treated with direct CAR T-cells or AdFITC-CAR T-cells.**

**(A)** Representative flow cytometry plots of live cells from BM single-cell suspensions at the terminal analysis of NSG mice engrafted with 5x10^6^ PID20 cells and seven days later injected i.v. with either 10^7^ direct anti-CD117 or direct anti-CD33 CAR T-cells (alone or 5x10^6^ each in combination) or 10^7^ AdFITC-CAR T-cells followed by i.p. administration of 12.5 μg CD33 and CD117 Db-FM, alone or in combination, at 12h intervals for three weeks.

**Supplementary Figure 13**


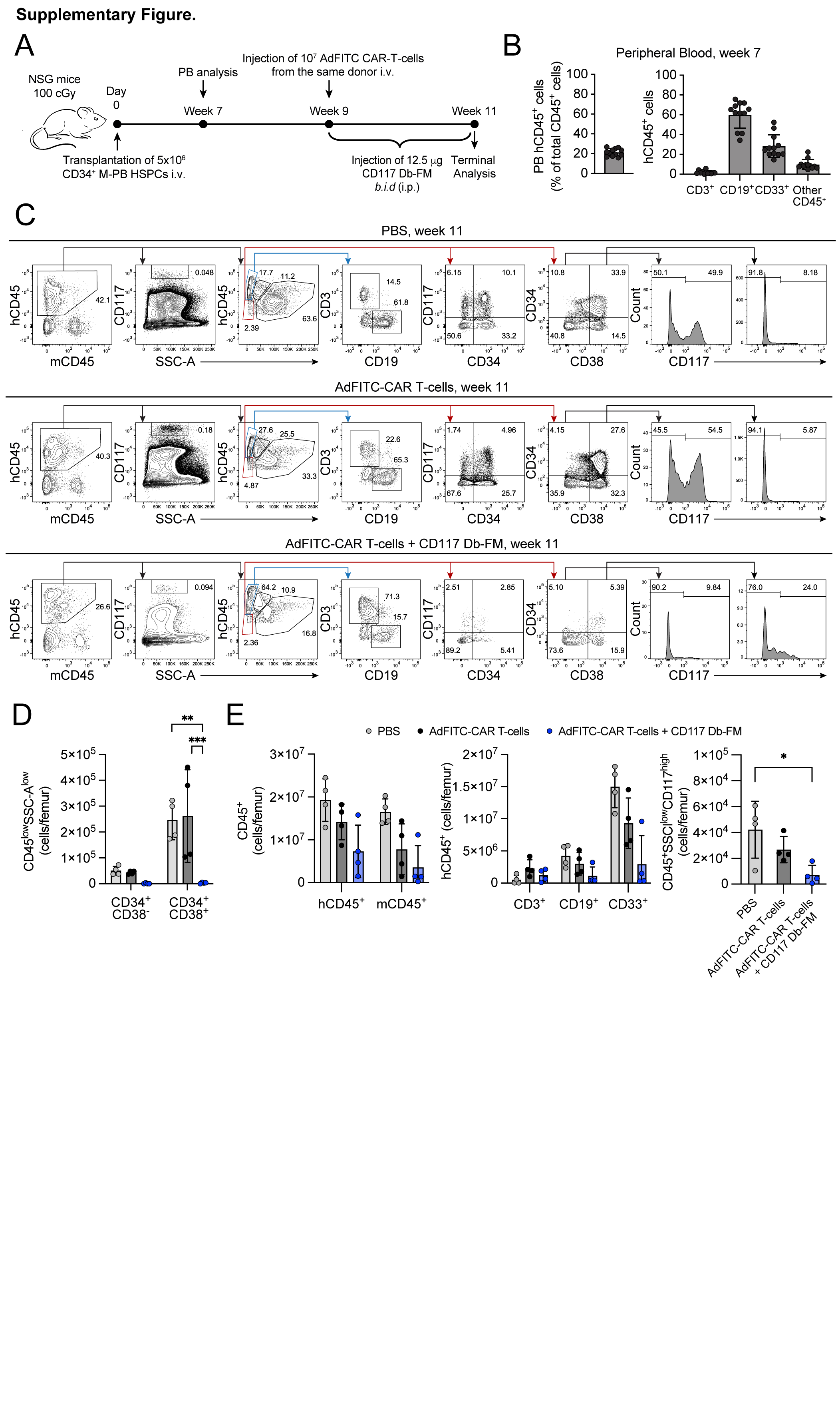


**Supplementary Figure 13. AdFITC-CAR T-cells in combination with CD117 Db-FM reduce healthy human CD117 expressing cells *in vivo*.**

**(A)** Schematic outline of the experimental setup. Sub-lethally irradiated NSG mice (100cGy) were engrafted i.v. with 5x10^6^ CD34-selected mobilized peripheral blood HSPCs (M-PB HSPCs). At week 7 after cell inoculation, human chimerism was assessed in peripheral blood by flow cytometry. Two weeks later, 10^7^ AdFITC-CAR T-cells, generated from the same M-PB HSPC donor, were injected i.v. with or without subsequent administration of 12.5 μg CD117 Db-FM i.p. every 12 hours for 10 days (n=4 mice per group). **(B)** Percentages of all human CD45^+^ cells (left) and human lineage antigen expression on cells (right) in peripheral blood 7 weeks after transplantation. Each symbol represents an individual mouse. **(C)** Representative flow cytometry plots of BM cells isolated from a single femur of a mouse at terminal analysis for each experimental group. Sequential gating is shown. **(D)** Absolute counts of human CD34^+^CD38^-^ and CD34^+^CD38^+^ progenitors of treated and untreated mice in the BM at terminal analysis. Statistical analysis was performed using one-way ANOVA; ** p <0.01, *** p <0.001. **(E)** Absolute counts of human and mouse CD45+ cells (left), CD3^+^/CD19^+^ lymphoid cells and CD33^+^ myeloid cells (middle) and CD45^+^SSC^low^CD117^high^ mast cells (right) in the BM at terminal analysis. Statistical analysis was performed using one-way ANOVA; * p <0.05.

Table S1.

**List of sample/patient characteristics**

| **#** | **Age** | **Sex** | **Diagnosis** | **Gene mutations** | **FISH / aCGH** | **Karyotype** | **CD45^dim^SSC^neg/low^ (% live cells) upon thawing** |
| --- | --- | --- | --- | --- | --- | --- | --- |
| 1 | 63 | M | AML | Not performed | n/a | 47,XY,+13[9]/46,XY[1] | 96.4 |
| 2 | 63 | M | AML | n/a | n/a | 42~45,XY,del(3)(p21), ?add(5)(q14).de\|(6)(q12q27),add(7)(q11.2), ?add(11)(q23),add(12)(p12), der(12;14)(q10;q10), -13, -16, -17, -18, -20, -21, -22, +1~4mar, inc[cp10] | 94.5 |
| 3 | 30 | M | AML | KIT | inv(16) | 46, XY, inv(16)(p13q22)[10/10] | 94.1 |
| 4 | 31 | M | Therapy asso. AML | FLT3, NRAS, KRAS | 11q cnLOH | 46, XY [20/20] | 89.4 |
| 5 | 65 | M | AML | ASXL1, IDH2, DNMT3A | none | 47,XY,+8[11] | 89.6 |
| 6 | 23 | M | AML | TP53 | none | 44-55.X,\|(Y)(q10),der(5)t(5:17)(q12,q21),der(7:18)(q11.2:q12),-8,der(8)t(8,15)(q2?3:q21),-9, add(12)(p12)mm5m6m7m8ner(21)t(18:21)(q22:q22),"3-13mar,lnc[cp12] | 97.8 |
| 7 | 45 | M | AML | NPM1A, NRAS | none | 46,XY[20] | 97.5 |
| 8 | 54 | F | MDS-EB2 | CBL, WT1, SF3B1 | n/a | 46,XX,inv(3)(q21q26),del(7)(p1?2)[cp3]/46,XX[19] | 23.2 |
| 9 | 49 | M | MDS-EB2 | NPM1A, DNMT3A | none | 46, XY[20] | 21.2 |
| 10 | 49 | F | AML | NPM1A, IDH1, PTPN11 | none | 46, XX[20] | 97.3 |
| 11 | 55 | M | AML | KIT | RUNX1-RUNX1T1 | 45,X,-Y,t(8;21)(q22;q22)[10] | 16.5 |
| 12 | 70 | M | AML (post CMML) | n/a | +21q | 46, XY, idic(21)(q22.3)[20] | 94.8 |
| 13 | 30 | M | AML | CEBPA, KIT, NRAS | none | 46,XY | 7.58 |
| 14 | 56 | F | AML | NPM1A, FLT3-ITD, DNMT3A, RAD21 | none | 46, XX | 95.2 |
| 15 | 56 | M | AML (post CMML) | SRSF2 | none | 46, XY | 88.9 |
| 16 | 77 | M | AML | ZRSR2, CEBPA, TET2 | none | 47,XY,+19[5]/46,XY[15] | 62.4 |
| 17 | 76 | F | AML | NPM1A, FLT3-ITD, DNMT3A | none | 46, XX | 94.5 |
| 18 | 49 | M | AML | WT1, CEBPA | none | 46, XY[20] | 93.1-95.9 |
| 19 | 54 | F | AML | CBFB-MYH1, FLT3, KIT | CBFB-MYH11 [t(16;16)] | 46, XX, t(16,16) (p13; q22) [19]/46, sl, del (7)(q31)[1] | 69.9 |
| 20 | 74 | F | AML | NPM1A, FLT3 | none | 46, XX[20] | 98.2 |
| Samples 1-17 were used in experiments depicted in Fig. S1B (surface immunophenotyping)  Samples 18-20 in Fig. 4 (*in vitro* experiments)  Sample 20 in Fig. 8, Fig. S11, Fig. S12 (*in vivo* experiments) | | | | | | | |

Table S2

**List of antibodies used in this study**

| **Target** | **Clone** | **Fluorochrome** | **Supplier** |
| --- | --- | --- | --- |
| hCD3 | UCHT1 | Brilliant Violet 711™ | Biolegend |
| hCD3 | OKT3 | PE, APC or Brilliant Violet 711 | Biolegend |
| hCD19 | SJ21C1 | PE | Ebiosciences |
| hCD25 | BC96 | Brilliant Violet 605™ | Biolegend |
| hCD33 | WM53 | PerCP-Cy5.5, PE or Brilliant Violet 711™ | Biolegend |
| hCD33 | PE67.6 | APC-Cy7 | Biolegend |
| hCD34 | QBEND-10 | FITC or PE | Thermo Fischer |
| hCD34 | 581 | PE-Cy7 | BioLegend |
| hCD34 | 581 | PE | BD Biosciences |
| hCD38 | HIT2 | Alexa Fluor™ 700 | Biolegend |
| hCD38 | HIT2 | APC | Biolegend |
| hCD45 | HI30 | eFluor™ 450 | Ebiosciences |
| hCD69 | FN50 | APC | Biolegend |
| hCD117 | 104D2 | PE-Cy7 | Ebiosciences |
| hCD117 | 104D2 | PE | Dako |
| hCD117 | 104D2 | APC | Biolegend |
| hCD123 | 6H6 | eFluor™780 | Thermo Fisher |
| hCD135 | BV10A4H2 | APC | Biolegend |
| hCD371 | 50C1 | Brilliant Violet 711™ | BD Biosciences |
| hCD371 | 50C1 | PerCP-Cy5.5 or APC | Biolegend |
| Anti-human lineage cocktail | n/a | FITC | Biolegend |
| mCD45 | 30-F11 | PE-Cy7 or Alexa Fluor™ 700 | Ebiosciences |
| mCD45 | 30-F11 | PerCP-Cy5.5 | Biolegend |
| Fluorescein | FIT-22 | APC | Biolegend |
